# The aldehyde (ALD) locus controls C6-aldehyde production in kiwifruit and affects consumer perception of fruit aroma

**DOI:** 10.1093/plphys/kiaf285

**Published:** 2025-06-30

**Authors:** Chunhui Huang, Lei Zhang, Xiuyin Chen, Tyler E McCourt, Tianchi Wang, Mindy Y Wang, Robert A Winz, John N McCallum, Samantha J Baldwin, Ross G Atkinson, Niels J Nieuwenhuizen

**Affiliations:** The New Zealand Institute for Plant and Food Research Limited (PFR), Auckland 1142, New Zealand; Agronomy College, Jiangxi Agricultural University, Economic and Technological Development Zone, Nanchang City, Jiangxi 330045, China; The New Zealand Institute for Plant and Food Research Limited (PFR), Auckland 1142, New Zealand; Hubei Key Laboratory of Germplasm Innovation and Utilization of Fruit Trees, Institute of Fruit and Tea, Hubei Academy of Agricultural Sciences, Wuhan 430064, China; The New Zealand Institute for Plant and Food Research Limited (PFR), Auckland 1142, New Zealand; The New Zealand Institute for Plant and Food Research Limited (PFR), Auckland 1142, New Zealand; The New Zealand Institute for Plant and Food Research Limited (PFR), Auckland 1142, New Zealand; The New Zealand Institute for Plant and Food Research Limited (PFR), Auckland 1142, New Zealand; The New Zealand Institute for Plant and Food Research Limited (PFR), Auckland 1142, New Zealand; The New Zealand Institute for Plant and Food Research Limited, Christchurch 8140, New Zealand; The New Zealand Institute for Plant and Food Research Limited, Christchurch 8140, New Zealand; The New Zealand Institute for Plant and Food Research Limited (PFR), Auckland 1142, New Zealand; The New Zealand Institute for Plant and Food Research Limited (PFR), Auckland 1142, New Zealand

## Abstract

Volatile C6-aldehydes contribute green/grassy notes to the aroma of many unripe fruits. C6-aldehydes are also likely important contributors to flavor intensity in fruits that remain green when ripe, including green-fleshed kiwifruit (*Actinidia* spp.). Here, we investigated the genetic basis for aldehyde production in kiwifruit in an *A. chinensis* mapping population. A major quantitative trait locus for producing multiple aldehydes was identified on chromosome 28 and named the aldehyde (ALD) locus. This locus co-located with 3 tandemly arrayed *A. chinensis LIPOXYGENASE* (*AcLOX4a–c*) genes in the Red5 genome. Expression of the ALD 13-LOX genes and aldehyde production decreased as the fruit developed and ripened in multiple *Actinidia* spp. *In planta* transient overexpression and biochemical analysis indicated that *AcLOX4a* and *AcLOX4c* produce hexanal and hexenal isomers. The third gene, *AcLOX4b*, was inactive and likely a pseudogene. The ALD LOX genes were targeted for CRISPR-Cas9 knockout, generating *A. chinensis* kiwifruit lines that contained insertions/deletions in all 3 target genes. Gas chromatography-mass spectrometry analysis showed that C6-aldehyde levels were reduced in leaves and fruit of the CRISPR-Cas9 lines, with a >90% reduction in line CAL5-2 compared with the control. Sensory aroma analysis showed that consumers could readily discriminate unripe CAL5-2 fruit from controls, describing the fruit as less grassy. Consumer discrimination was weaker in ethylene-ripened CAL5-2 fruit, likely due to high levels of fruity esters. Our results validate the importance of C6-aldehydes in kiwifruit flavor, and our characterization of the ALD locus is a critical step toward maintaining and improving flavor intensity in kiwifruit.

## Teaser

CRISPR-Cas9 knockouts of *LIPOXYGENASE* genes at the aldehyde (ALD) locus in kiwifruit reduce C6-ALD production and affect consumer perception of fruit flavor intensity.

## Introduction

Fruit flavor is an important driver for consumer liking and is considered a combination of taste perceived in the mouth and aroma detected in the nose. Fruit flavor is driven by the balance of sugars, acids, and volatile organic compounds (VOCs) (Colantonio et al. 2022). The VOCs, especially, are responsible for the characteristic flavor of each fruit variety. In tomato, over 400 VOCs have been detected, with 25 to 30 compounds contributing substantially to the flavor of the fruit (Tieman et al. 2017; Klee and Tieman 2018; Kaur et al. 2023). In strawberry, a similar number of volatiles are associated with flavor intensity and consumer acceptance (Schwieterman et al. 2014; Ulrich and Olbricht 2016). Fruit VOCs such as esters, terpenes, and phenylpropenes are derived from multiple precursors and via different pathways (El Hadi et al. 2013). Aldehydes contribute green-grassy notes to fruit aroma and are derived from fatty acids and branched chain amino acids. Aldehydes are often found at high levels in unripe fruit, and their concentrations typically decrease in ripe fruit, whereas esters are the predominant aroma component and proposed to be a predictive signal to frugivores for sugar content in ripe fruit (Nevo et al. 2022).

**Figure 1.**
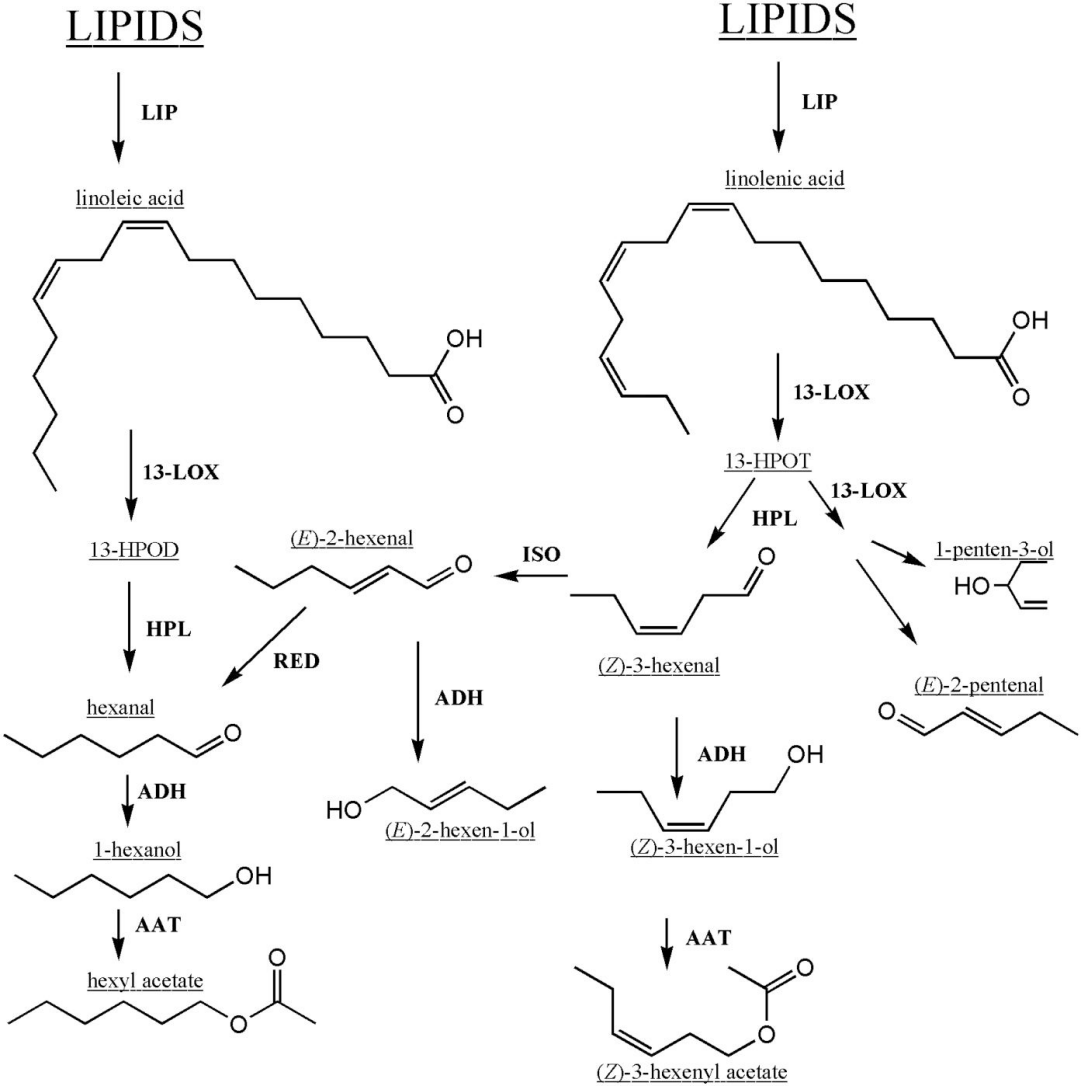
The LOX pathway involved in the production of VOCs in fruit. Major chemicals are underlined, and enzymes are highlighted in bold. HPOD/T, 13(*S*)-hydroperoxy-9(*Z*),11(*E*)-octadecadienoic acid/13(*S*)-hydroperoxy-9(*Z*),11(*E*),15(*Z*)-octadecatrienoic acid; LIP, lipase; 13-LOX, 13-lipoxygenase; HPL, hydroperoxide lyase; ISO, (*Z*)-3:(*E*)-2-hexenal isomerase; RED, hexenal reductase; ADH, alcohol dehydrogenase; AAT, alcohol acyl transferase.

The biosynthesis of the C5- and C6-aldehydes and their derivatives occurs predominantly through the lipoxygenase (LOX) pathway (Feussner and Wasternack 2002) (Fig. 1). Inside chloroplasts, free unsaturated fatty acids (FFAs) are first released from triacylglycerols (TAGs). Lipases fulfil this biological role by catalyzing the hydrolysis of TAGs to yield FFAs, diacylglycerols (DAGs), monoacylglycerols (MAGs) and glycerol. Recently, the *Solanum lycopersicum lipase 8* gene (*Sl-LIP8*) was shown to be responsible for cleavage/release of 18:2 and 18:3 acyl groups from glycerolipids in tomato (Li et al. 2020). Lipoxygenases are nonheme iron dioxygenases that catalyze the formation of corresponding hydroperoxides from the released polyunsaturated fatty acids (PUFAs), such as linoleic (C18:2) and linolenic acid (C18:3). These 2 PUFAs become the substrate for 13-LOX enzymes, which catalyse the oxygenation at position 13 of the carbon chain (in contrast to the 9-LOX enzymes). The 13-hydroperoxides produced by the 13-LOX enzymes are subsequently cleaved by the enzyme hydroperoxide lyase (HPL) to release the C6-aldehydes from its C18-precursors. The C6-aldehydes can be converted to their corresponding alcohols by the action of alcohol dehydrogenases (Speirs et al. 1998). Subsequently, these C6-alcohols can be converted into esters such as hexyl- and hexenyl acetate by alcohol acyl transferases (AATs) (Souleyre et al. 2014; Zhou et al. 2021). Alternatively, unsaturated C6-aldehydes can be isomerized by the enzyme (*Z*)-3:(*E*)-2-hexenal isomerase (Kunishima et al. 2016) or reduced to hexanal by reductases (Tanaka et al. 2018) (Fig. 1).

LOX genes have been identified from multiple fruit and their expression has been linked with the production of aldehydes (Pérez et al. 1999; Weichert et al. 2002; Contreras and Beaudry 2013). Most known plant LOXs show stereospecificity and synthesize (*S*)-hydroperoxides as the predominant epimers (Grechkin 1998) but in apple, recombinant MdLOX1a and -2a formed 13(*R*)-hydroperoxides as major products (Schiller et al. 2015). Suppression of genes involved in aldehyde production in fruit has been described exclusively in tomato to date. Downregulation of *TomloxC* expression by antisense or co-suppression (Heitz et al. 1997; Chen et al. 2004), resulted in a 98% reduction in fruit C6-volatiles, including hexanal, and isomers of hexenal and hexenol, while leaf C6-volatiles were also reduced in some lines (Chen et al. 2004), while antisense suppression of *TomloxA* and *B* didn’t affect flavor volatile levels (Griffiths et al. 1999). Later work showed that levels of aromatic C5-volatiles, such as 1-penten-3-ol/one and pentanal, were also significantly reduced in *TomloxC* suppressed plants (Shen et al. 2014). Plants suppressed for *LeHPL* showed an increase in aromatic C5-volatile concentrations, presumably due to increased 13-hydroperoxy substrate availability, that could be further metabolized by TomloxC (Shen et al. 2014), while in potato suppression of *HPL* showed a similar response with increased C5-volatiles/reduced C6-volatiles (Vancanneyt et al. 2001). Downstream of the LOX and HPL step, modification of *alcohol dehydrogenase 2* levels in tomato has also been shown to influence the balance between some of the C6-aldehydes and the corresponding C6-alcohols such as 1-hexanol and (*Z*)-3-hexenol, associated with flavor in ripening tomato fruit (Speirs et al. 1998). Outside of fruit, in legumes, CRISPR knockout editing of 3 LOX genes (*GmLox1*-*3*) resulted in soybeans (*Glycine max* L.) completely devoid of 13-LOX activity (Wang et al. 2020), while in yellow peas (*Pisum sativum* L.), CRISPR LOX editing led to major changes in fatty acid and volatile profiles (Bhowmik et al. 2023).

Kiwifruit (*Actinidia* spp.) flavor is characterized by a combination of the green/grassy notes from unsaturated fatty acid derived C6-compounds, especially hexanal and *(E)*-2-hexenal together with fruity esters such as methyl- and ethyl butanoate (Lan et al. 2021). Other compounds such as the terpenes 1,8-cineole and terpinolene derived from terpene synthase enzymes (Wang et al. 2023), as well as dimethyl sulfide (DMS) contribute to the characteristic blends of VOCs underlying each kiwifruit cultivar (Nieuwenhuizen et al. 2015; Zeng et al. 2020). The world market is dominated by production of the green-fleshed *Actinidia chinensis* var. *deliciosa* cultivar “Hayward,” which has an acidic taste and a pronounced green/grassy aroma, while a small section of the green-fleshed market space is occupied by grape-sized kiwiberries from *A. arguta*, that are described as having tropical, fruit candy, and green flavors (Garcia et al. 2012). In the last 2 decades, yellow- and red-fleshed *A. chinensis* var. *chinensis* cultivars have become popular, with more tropical flavors and less green/grassy notes (Patterson et al. 2003; Wang et al. 2003, 2011; Hernández and Craig 2015).

Genetic studies have revealed the importance of terpene synthases and AATs to volatile terpene and ester production, respectively, and the importance of these compounds to kiwifruit flavor (Nieuwenhuizen et al. 2015; Zeng et al. 2020; Souleyre et al. 2022). However, the genetics underpinning aldehyde production in kiwifruit are less clear. The expression of LOX genes in “Hayward” fruit has been reported previously (Zhang et al. 2006; Zhang et al. 2009a, 2009b). Six partial LOX gene sequences were identified and named *AdLOX1–6*. The proposed 9-LOX genes *AdLOX1* and *5* (*type 1*) showed increased expression as fruit ripened, while the proposed 13-LOX genes/(*type 2*) *AdLOX2–4* and *6* showed reduced expression (Liavonchanka and Feussner 2006; Zhang et al. 2009a). *AdLOX2–4* and *6* expression correlated with a reduced C6-aldehyde levels (hexanal/(*E*)-2-hexenal) observed during ripening. Changes in C6-aldehyde levels and LOX gene expression were affected by 1-MCP treatment, suggesting involvement of ethylene in suppressing the LOX pathway during ripening (Zhang et al. 2009a).

Green grassy aroma notes are not just important in savory fruits such as tomato, olive, and cucumber (Aparicio and Morales 1998; Weichert et al. 2002). In sweet-type fruit such strawberry, aldehydes and alcohols such as hexanal, (*E*)-2-hexenal and (*E*)-3-hexen-1-ol are important for the green, unripe notes in strawberry aroma and their production is cultivar and ripening stage dependent (Jetti et al. 2007). In peach fruit, C6-aldehydes and alcohols also contribute green-note aromas that are balanced with lactone and ester fruity notes (Wang et al. 2009), while “Granny Smith” apples have distinct green-grassy aromas, mainly C6-alcohols and derived esters, associated with sensory green character and derived from the LOX pathway (Rowan et al. 1999; Hongsoongnern and Chambers 2008). To identify potential genetic determinants for aldehyde production in kiwifruit, another sweet-type fruit, we employed a genetics approach using a segregating mapping population and functionally characterized candidate LOX genes by transient overexpression in *Nicotiana benthamiana*. CRISPR-Cas9 knockout lines for key LOX genes allowed production of fruit with much reduced levels of key volatile aldehydes for consumer sensory analysis. Our sensory results directly validate the importance of C6-aldehydes to kiwifruit flavor and how C6-aldehydes are likely to contribute to fruit flavor in other species.

## Results

### Aroma volatiles in kiwifruit at harvest and when eating ripe

**Figure 2.**
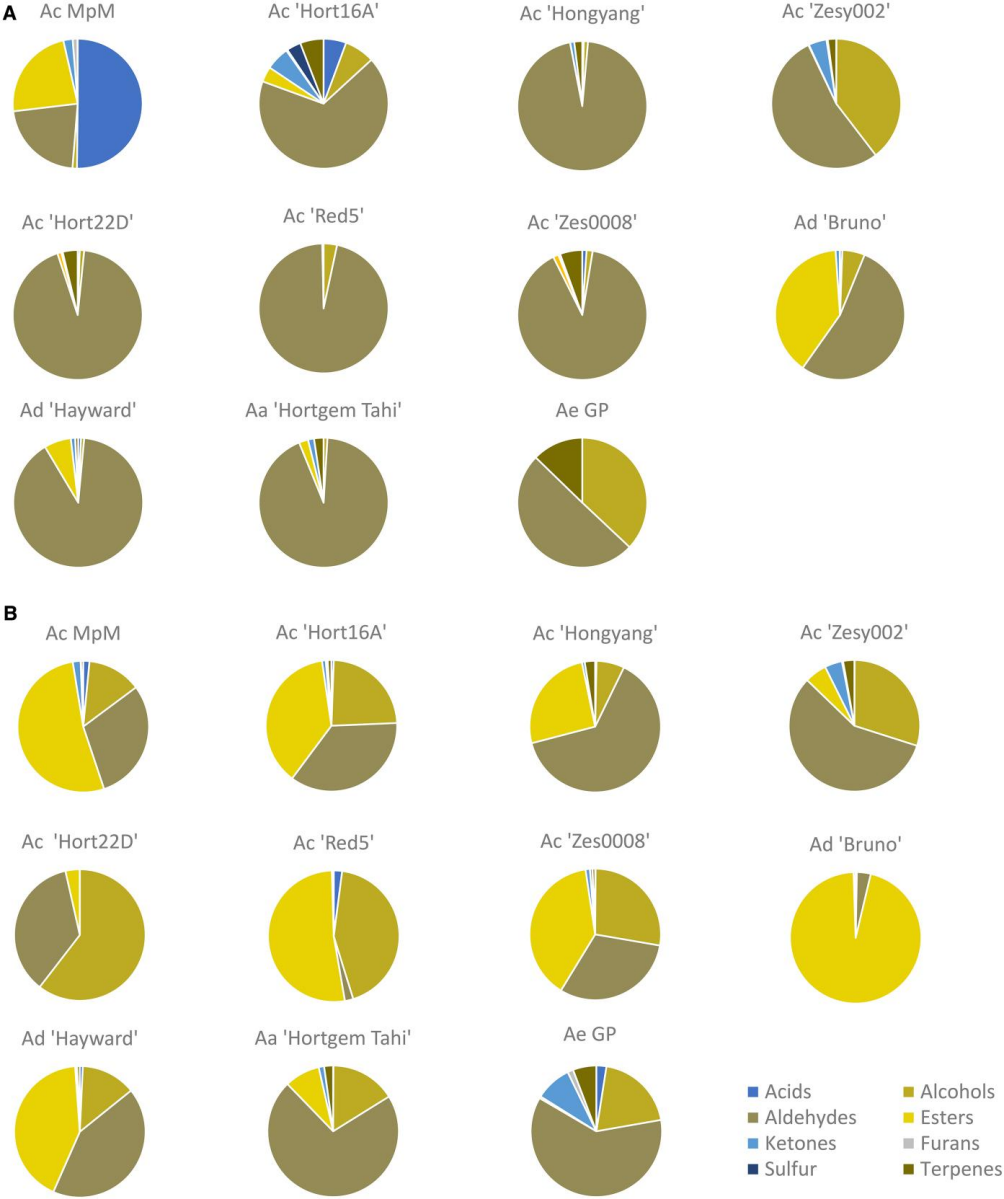
Fruit volatile composition of 11 kiwifruit cultivars. Concentrations were measured at the commercial harvest **A)** and eating ripe **B)** stage, based on 3 biological replicates. Each volatile is presented as a fraction of the total and full data are presented in Supplementary Table S1. Species: *Actinidia chinensis* var. *chinensis* (Ac), *A. chinensis* var. *deliciosa* (Ad), *A. arguta* (Aa) and *A. eriantha* (Ae). The genotype MpM is described in Fraser et al. (2007) and genotype GP in Prakash et al. (2017).

Volatile production was measured by GC-MS in 11 kiwifruit cultivars/accessions representing the major commercial *Actinidia* fruiting species (*A. chinensis*, *A. arguta* and *A. eriantha*). Large shifts in the volatile composition were observed in all fruit as they progressed from harvest to eating ripe. At harvest, the profiles were dominated by the presence of C6-aldehydes (Fig. 2A; Supplementary Table S1A). At the eating ripe stage, while aldehydes remained abundant in many cultivars, the profiles typically showed increasing concentrations of esters and alcohols (Fig. 2B; Supplementary Table S1B).

### Volatiles produced during fruit development and ripening in 4 kiwifruit genotypes

**Figure 3.**
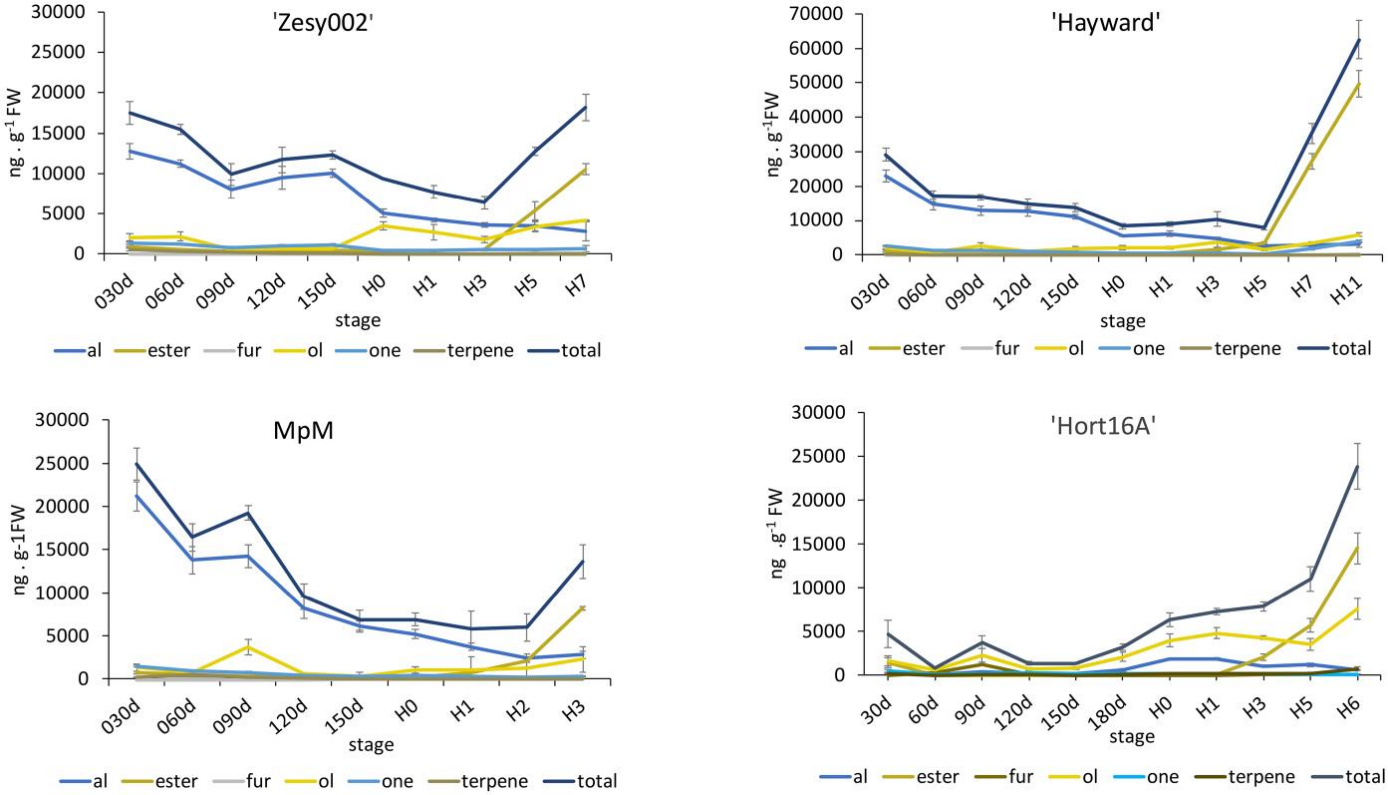
Changes in headspace volatile aroma production during fruit development and ripening in 4 *A. chinensis* genotypes. The genotypes “Zesy002”, “Hayward”, MpM (Fraser et al. 2007) and “Hort16A” were analysed. Volatile compound classes: aldehydes (blue), esters (brown), furans (gray), alcohols (yellow), ketones (light blue), terpenes (dark gray) and total volatiles (dark blue). Fruit development was sampled monthly from 30 days (30 d) after full bloom. Fruit at commercial harvest (H0) were treated with ethylene and samples collected at 1–11 days later (H1–H11). Data are mean ± SE from 3 biological replicates and full data are presented in Supplementary Table S2.

To gain further insight into how production of C6-aldehydes was regulated during fruit development, samples from 4 *A. chinensis* genotypes were harvested monthly, and HS VOCs were analyzed by GC-MS. At harvest, fruits were treated with ethylene and VOC production was monitored until the fruit became overripe (H0-H11) (Fig. 3). Total volatile concentrations declined during fruit development in 3 of the 4 cultivars tested. Total aldehyde concentrations (mostly hexanal and the hexenal isomers, Supplementary Table S2) declined in the same pattern, and typically represented the majority of VOCs during fruit development. Aldehyde levels declined further post-harvest, while alcohol and ester concentrations increased as the fruit softened. Alcohols and esters become the most abundant volatiles during the late autocatalytic ethylene ripening phase. In “Hayward” fruit, ester concentrations were found in much higher quantities at the very ripe/overripe stages, compared with “Zesy002”, MpM and “Hort16A”. Ketone, furan, and terpene concentrations were generally detected at low levels throughout fruit development and ripening.

### Quantitative trait loci mapping of volatile C5/C6-aldehydes in a segregating *A. chinensis* population

To investigate the genetic basis for aldehyde production in kiwifruit, volatile production was investigated in an intraspecific, diploid *A. chinensis* mapping population (AcMPO) (Fraser et al. 2009). The parents were selected for their diversity of fruiting characters, with the those of the male being inferred from the attributes of female siblings. AcMPO consists of 272 plants of which approximately half are female and bear fruit. Volatile datasets were obtained on eating ripe fruit using 2 methods, headspace volatile trapping (HS) and extraction into organic solvent (SE), followed by GC-MS analysis. Fruit was harvested at 2 week intervals over a 3-month period to account for differences in time to fruit maturity between genotypes. In the HS analysis, 18 compounds were selected for mapping, based on abundance and frequency of detection. These compounds included 8 esters and 6 C5/C6-aldehydes (Supplementary Table S3A). The 2 most abundant HS compounds were the esters ethyl butanoate and methyl butanoate, which were detected in all the mapping population members, and together accounted for ∼66% of total VOCs at the eating ripe stage (Supplementary Table S3A). Ethanol was the most abundant alcohol, with a mean concentration of ∼1000 ng·g^−1^ fresh weight (FW). It was also detected in all AcMPO members, representing ∼9% of HS VOCs (Supplementary Table S3A). (*E*)-2-hexenal was the main aldehyde, followed by hexanal. Together these 2 aldehydes accounted for >99% of the C5/C6-aldehydes and derived compounds and represented ∼24% of mean VOCs in the AcMPO (Supplementary Table S3A). The terpene cymene was detected in small quantities, averaging 14 ng·g^−1^ FW. In the solvent extraction (SE) analysis, 70 volatiles were quantified. Again, ethyl and methyl butanoate accounted for the majority of the volatiles (64%), while (*E*)-2-hexenal and hexanol accounted for ∼8% of total VOCs (Supplementary Table S3B). Other abundant compounds included methyl- and ethyl benzoate and butyl butanoate, as well as benzyl alcohol, representing 6.4/3.0/4.6/2.7% of mean VOCs respectively.

**Figure 4.**
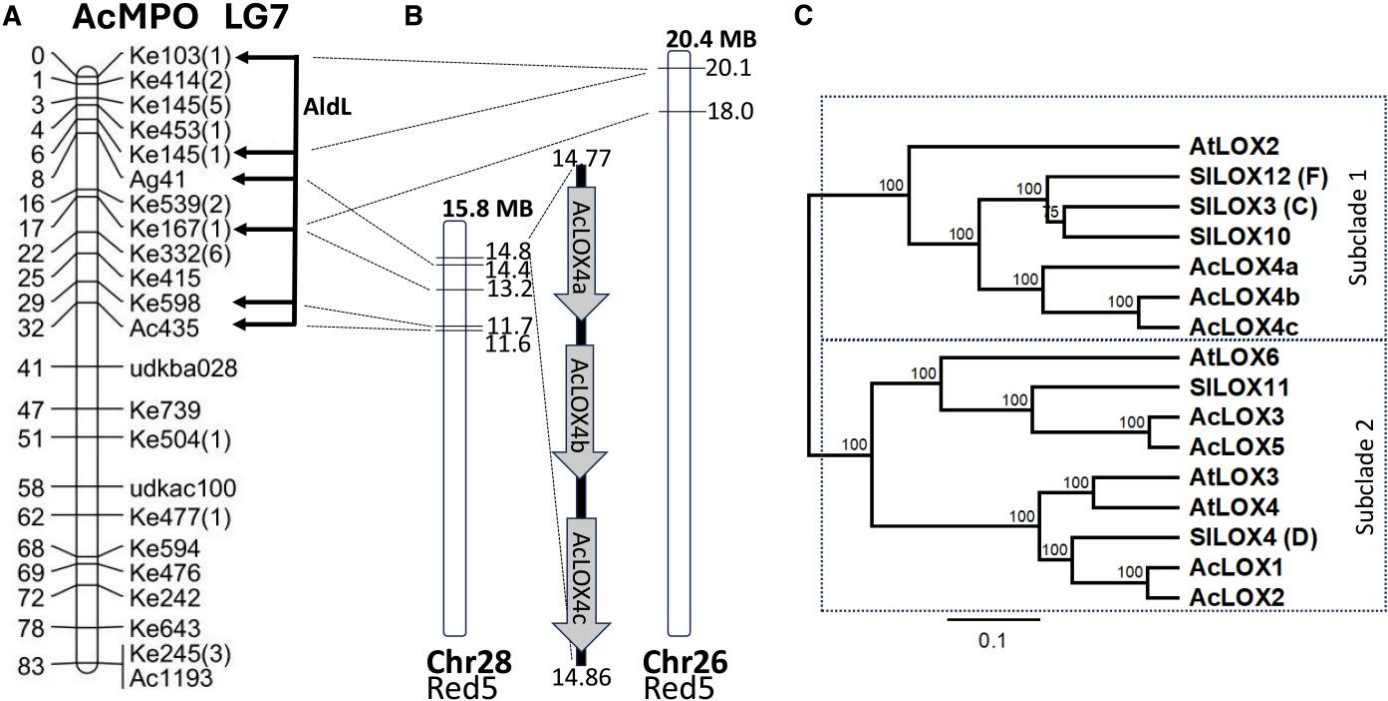
Mapping and phylogenetic analysis of the ALD locus and 13-LOX genes in kiwifruit, Arabidopsis and tomato. **A)** LG 7 markers linked with C6-aldehyde and alcohol production at the ALD locus in the mapping population AcMPO. **B)** Physical location of the linked markers on chromosomes (Chr) 26 and 28 and the corresponding location of the *AcLOX4a*, *b* and *c* genes on chromosome 28 at ∼14.8 megabase (MB) in Red5. **C)** Phylogram of the evolutionary relationship between *Actinidia chinensis* (Ac), *Arabidopsis thaliana* (At) and tomato (*Solanum lycopersicum*, Sl) type 2 13-LOX genes. C, D, F are tomato *Tomlox* C, D, and F respectively. Scale bar in **C)**: number of substitutions divided by the length of the sequence. Distinct 13-LOX types/subclades have been observed among other angiosperm species (Camargo et al. 2023). Bootstrap values are based on 1,000 iterations.

Analysis of the volatile data was performed using ANOVA and putative quantitative trait loci (QTL) were identified that were associated with the production of individual esters, alcohols, aldehydes, and terpenes (Supplementary Table S3). A QTL affecting multiple ester compounds that has been previously investigated (Souleyre et al. 2022) was associated with markers Ke714, Ac1340, and Ac661 on LG3. Production of the terpene cymene was strongly associated with several markers on the bottom of LG22 centered around the 66 to 78 cM interval (Supplementary Table S4), in both the HS and SE datasets. A large set of markers associated with C5- and C6- aldehydes and related alcohol compounds (including (*E*)-2-hexenal, hexanal, (*E*)-2-hexenol, 1-hexanol, and 1-penten-3-ol) were identified in both the HS and SE datasets, and with untransformed as well as with log10 transformed data analysis (Supplementary Table S4). The markers most significantly associated with these compounds were all located near the top of LG7, between 0 and 32 cM and centered around marker Ke167(1) at 17 cM (Fig. 4A). A detailed QTL interval mapping analysis was performed by Hayley Knott regression using 1,000 permutations for (*E*)-2-hexenal, hexanal, (*E*)-2-hexenol and 1-penten-3-ol (Haley and Knott 1992). Only the former 2 showed significant QTL intervals based on 1.5 LOD (logarithm of the odds) support intervals (Supplementary Fig. S1), while (*E*)-2-hexenol and 1-penten-3-ol were not significant (both around ∼1 LOD above the LOD threshold).

By comparing the genetic location of the markers to their corresponding genome location, (Fig. 4, A and B; Supplementary Tables S5 and S6), 2 genomic candidate regions were identified based on the Red5 *A. chinensis* var. *chinensis* genome sequence (Pilkington et al. 2018). Using BLASTN searching with the respective marker primer sequences (Supplementary Table S5A), the genetic markers Ke103(1), Ke145(1), and Ke167(1) located to the top of chromosome (Chr)-26 (18 to 20.1 mb), while the genetic region spanning from Ke167(1) to Ac435 located to the top of Chr 28 (11.6 to 14.4 mb) (Fig. 4B; Supplementary Table S5B).

### Three LOX genes co-locate with the ALD locus

A search for LOX pathway-related LOX and HPL genes in the *A. chinensis* Red5 genome identified 14 LOX genes (seven 13-LOX; seven 9-LOX) and 2 HPL genes (Supplementary Tables S5 and S6). A complex cluster of 3 tandemly arrayed LOX gene models (Acc32486/87/88) co-located with the segregating markers for aldehyde production on LG7 and the corresponding regions on Chr 26 and 28. The 3 gene models for *AcLOX4a*, *b* and *c* were located between 14.77 and 14.86 mb on Chr 28 in close proximity to the markers Ke167 and Ag41 (Fig. 4B; Supplementary Tables S5 and S6). This cluster of 3 genes was designated the Aldehyde locus (ALD). None of the other HPL or LOX genes present in the *A. chinensis* genome (Supplementary Table S6) were located on Chr 26 or Chr 28, apart from *AcLOX10* (a 9-LOX), which was located at the center of Chr 28 (at ∼7.8 mb) outside of the ALD locus region (Fig. 4B; Supplementary Table S5). The AcLOX4a–c proteins cluster together with *AtLOX2* and *SlLOX3* (*TomloxC*) in subclade 1 (Fig. 4C) that have previously been reported to impact C5/C6-aldehyde production in Arabidopsis and tomato respectively (Chen et al. 2004; Shen et al. 2014; Mochizuki et al. 2016).

Within the candidate regions on Chr 26 and 28, a total 517 gene models were identified. In addition to the 3 aforementioned LOX gene candidates, another eleven putative lipid metabolism-related genes were identified (Supplementary Table S7—highlighted in yellow). These genes included a phospholipase gene (Acc32440) and 2 enoyl-[acyl-carrier-protein] reductases (Acc30428, Acc32493) (Supplementary Table S7).

### Expression of *AcLOX4a–c* genes during kiwifruit development and ripening

**Figure 5.**
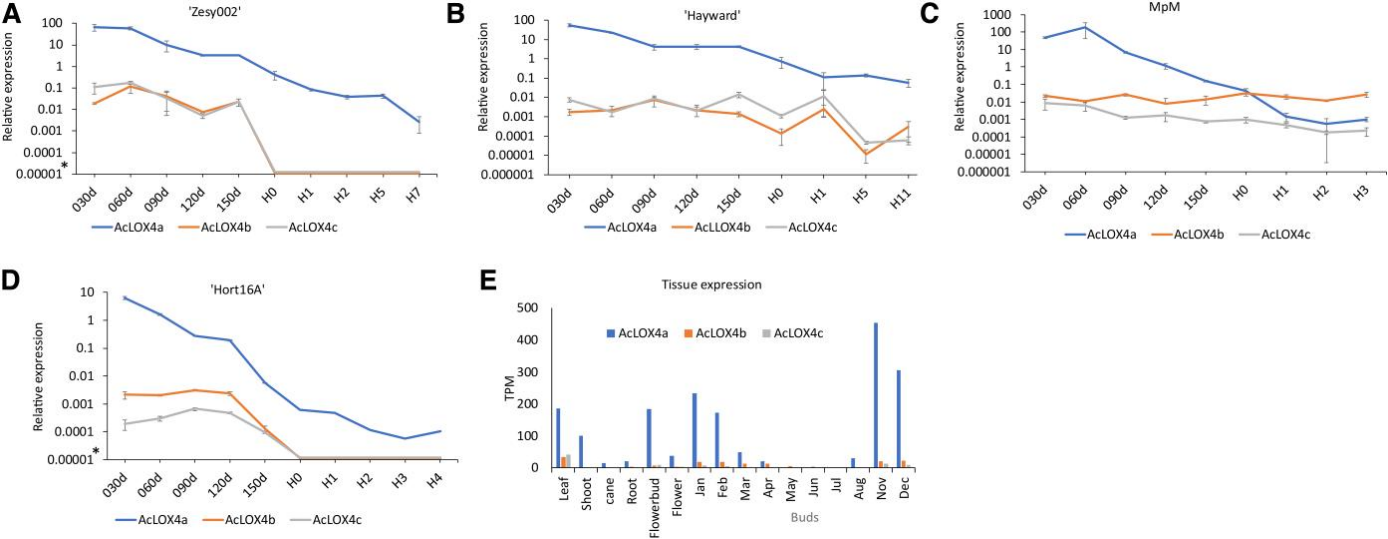
*AcLOX4a–c* relative gene expression in 4 kiwifruit genotypes. Gene expression was analysed during fruit development and ripening **A–D)** and in various “Hort16A” tissues **E)**. Expression was measured by RT-qPCR **A–D)** using gene-specific primers and normalized relative to the geometrical mean of 2 reference genes (Supplementary Table S8) or by RNAseq (“Tissue expression” **E**)—see also Supplementary Table S9B). *: samples with a threshold cycle of ≥40 in RT-qPCR were considered not expressed as visualized in the graphs at 0.00001. TPM, Transcripts per million reads. Fruit development was sampled from 30 days (30 d) after full bloom at monthly intervals. Fruit at commercial harvest (H0) were treated with ethylene and samples collected 1–11 days later (H1–H11). Tissue expression was sampled in various tissues including buds from January to December. *AcLOX4a*: blue line/bar *AcLOX4b*: orange line/bar, *AcLOX4c*: gray line/bar. Data are mean ± SE from 3 biological replicates.

Expression of the 3 LOX genes (*AcLOX4a–c*) located at the ALD locus was investigated in detail by RT-qPCR in 4 *A. chinensis* genotypes (Fig. 5). Primers (Supplementary Table S8) for each gene were shown to be gene-specific even though they shared >81% nucleotide identity excluding gaps (Supplementary Fig. S2, A to D). *AcLOX4a* showed the highest level of expression throughout fruit development until harvest. *AcLOX4a* expression was highest at 30 to 60 days after pollination and declined steadily over fruit development until harvest and declined further post-harvest. *AcLOX4a* expression was ∼1000-fold higher than that of *AcLOX4b* and *4c* (Fig. 5). MpM is the only genotype where expression of *AcLOX4b* was higher than *AcLOX4a* from stage H2 onwards. The declining expression pattern of *AcLOX4a* expression correlates positively (Pearson *R* = 0.52, Supplementary Table S9A) with the reduction in total aldehyde production during fruit development and post-harvest (Figs. 2 and 3 vs Fig. 5). *AcLOX4b* and *AcLOX4c* expression correlated only weakly with total aldehyde abundance (*R* = 0.28 to 0.29, Supplementary Table S9A). Interestingly, expression levels of *AcLOX4a* in “Hort16A” were >10-fold lower than in the other 3 genotypes at the same developmental stage. This fits with the GC-MS data presented in Supplementary Table S2, which showed lower aldehydes in Hort16A” during all stages of fruit development and previously published data (Garcia et al. 2012) that reported that “Hort16A” produces 250-fold less (*E*)-2-hexenal and 25-fold less hexanal compared with “Hayward” at the ripe fruit stage. In both “Hort16A” and “Zesy002”, *AcLOX4b* and *4c* were not detected in any of the harvest stages (threshold cycle in RT-qPCR ≥40) (Fig. 5). In “Hort16A”, *AcLOX4a* was also expressed in a range of other tissues including leaf, shoot, and buds (Fig. 5, lower panel). Expression of *AcLOX4b, c* was lower than *AcLOX4a* in all sampled tissues.

### Transient overexpression of *AcLOX1–4*

**Figure 6.**
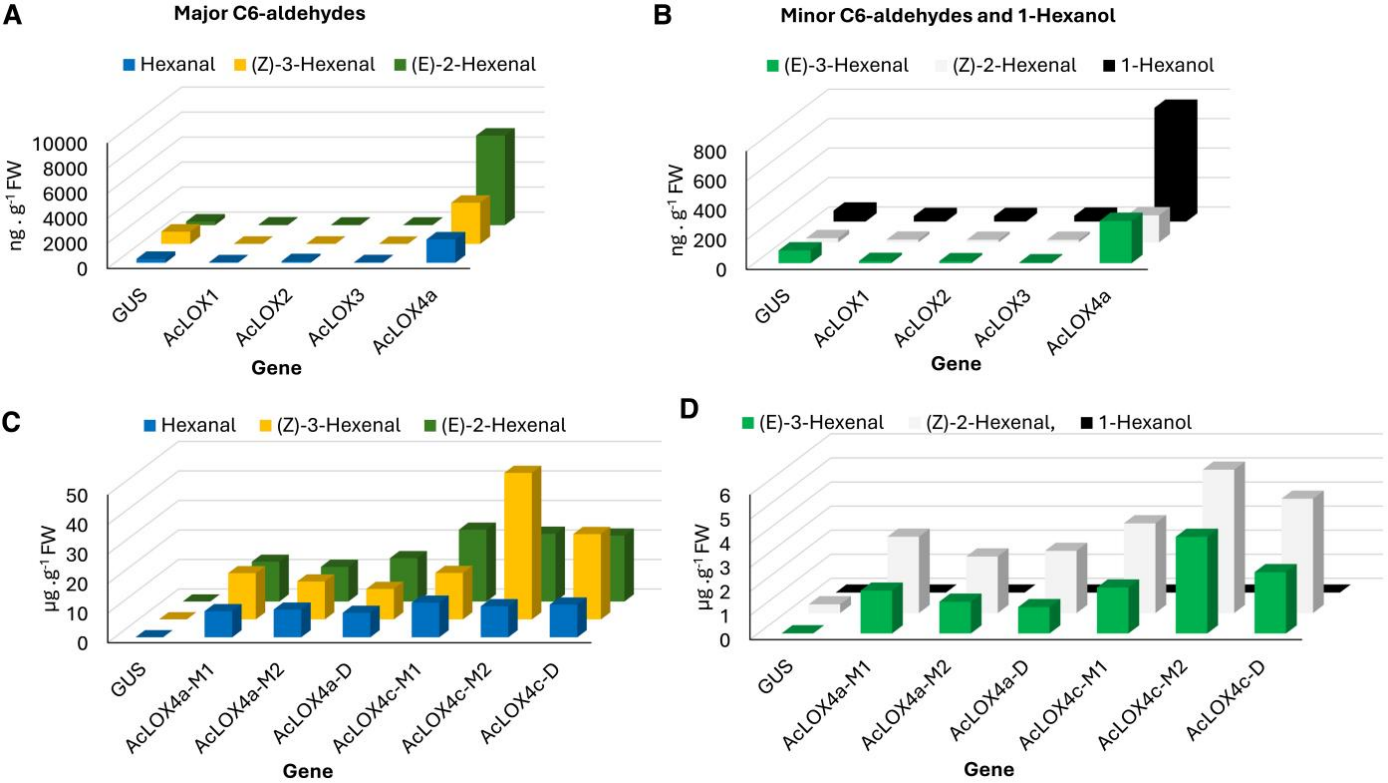
C6-volatiles produced by overexpression of *A. chinensis* 13-LOX genes in *N. benthamiana* leaves and measured by SPME GC-MS. **A)** Major leaf C6-aldehydes from *AcLOX1–4*, **B)** minor C6-aldehydes and alcohol. **C)** Major and **D)** minor C6-aldehydes and alcohol produced in *N*. *benthamiana* by *AcLOX4a* and *AcLOX4c* alleles derived from the mapping population mother (M1, M2) and father (D1). Bars represent means based on 3 biological replicates and GUS represents the negative control.

To test whether any of the 13-LOX type family members *AcLOX1–5* can contribute to C5/C6-volatile production *in planta*, primers were designed (Supplementary Table S5) to amplify their open reading from ripe MpM fruit cDNA. *AcLOX1–4* were successfully amplified, but *AcLOX5* (Supplementary Table S5) was not. *AcLOX1–4* were then transiently overexpressed in *Nicotiana benthamiana* and extracts were assayed for volatile production using solid phase micro extraction (SPME) followed by GC-MS analysis. The results indicated that *AcLOX4*, but not *AcLOX1–3*, produced markedly elevated levels of C6-aldehydes, including the 4 hexenal isomers and hexanal, compared with the pHEX2-GUS negative control (Fig. 6, A and B). AcLOX4a overexpression resulted in elevated concentrations of (*E*)-2- and (*Z*)-3-hexenal (25- and 10-fold respectively) and hexanal (3.3-fold) as well as smaller increases in (*E*)-3- and (*Z*)-2-hexenal and 1-hexanol compared with GUS control. *AcLOX1*, *2* and *3* did not show any increases of C6-aldehydes or C6-alcohols.

To investigate the relative contribution of the *AcLOX4a–c* genes to aldehyde production in AcMPO, alleles from both the female (MpM) and male parent (MpD) were cloned. For MpD, one allele each of *AcLOX4a* (4a-D1) and *AcLOX4c* (4c-D1) were amplified successfully multiple times. For MpM, 2 alleles each for *AcLOX4a* (4a-M1, 4a-M2) and *AcLOX4c* (4c-M1, 4c-M2) were cloned. No full-length cDNA corresponding to *AcLOX4b* was amplified. The gene model for *AcLOX4b* (Acc032487) is truncated in the Red5 genome assembly (Supplementary Fig. S2, A and B), and although RT-qPCR suggests it is expressed at low levels in all cultivars tested (Fig. 5), it likely represents an inactive gene/pseudogene, missing the N-terminus. A detailed summary of the allele analysis is listed in Supplementary Table S10. The 6 cloned *AcLOX4a* and *c* alleles from AcMPO were transiently overexpressed in *N. benthamiana* leaves and all 6 alleles were active and produced C6-aldehydes at similar concentrations (Fig. 6, C and D).

### Biochemical analysis of AcLOX4a and AcLOX4c

Transient *in planta* expression showed that both *AcLOX4a* and *AcLOX4c* genes from both parents could produce elevated C6-aldehyde levels at similar ratios and amounts. To compare the enzymatic properties of AcLOX4a and AcLOX4c, the 2 alleles amplified from MpD (AcLOX4a-D1 and AcLOX4c-D1) were expressed as maltose binding protein (MBP) fusions in *E. coli* and purified to near homogeneity. Kinetics analysis showed that both enzymes were active, using both α-linolenic acid (ALA) and linoleic acid (LA) as substrates (Table 1; Supplementary Fig. S3). The apparent k_m_ values for linoleic acid showed that AcLOX4a-D1 had a ∼3-fold higher K_m_-app compared with AcLOX4c-D1. More similar values were observed when considering α-linolenic acid as substrate (132 vs 76 µM respectively). In contrast, the V_max_-app for AcLOX4a-D1 was between 37- and 135-fold higher than for AcLOX4c-D1. Overall, the enzymatic efficiency of AcLOX4a-D1 (K_cat_/K_m_) was also much higher for AcLOX4a-D1. Inhibition for both enzymes was observed at high substrate concentrations (Table 1; Supplementary Fig. S3C), with the AcLOX4a-D1 enzyme being more active at moderate to high substrate concentrations, compared with AcLOX4c-D1.

**Table 1.** Kinetic parameters of the 13-LOX enzymes AcLOX4a-D1 and AcLOX4c-D1 using linoleic acid and α-linolenic acid as substrates

Substrate	Enzyme	V_max_-app	K_m_-app	k_cat_	K_cat_/K_m_	Inhibition
		(nmol.s^−1^.mg^−1^)	(µM)	(s^−1^)		(µM)
Linoleic acid	AcLOX4a-D1	382.2	193.2	54.0	0.279	>125
Linoleic acid	AcLOX4c-D1	2.8	59.2	0.4	0.007	>50
α-linolenic	AcLOX4a-D1	139.3	132.8	19.7	0.148	–
α-linolenic	AcLOX4c-D1	3.8	75.8	0.5	0.007	>100

Values are derived from nonlinear regression fit using the Michaelis Menten equation using the Origin 2022 software (Supplementary Fig. S3). The apparent (app) K_m_ and V_max_ terms are used to indicate that the 13-LOX enzymes may differ in iron load and display signs of substrate inhibition at high substrate concentrations.

### CRISPR editing of LOX genes at the ALD locus

**Figure 7.**
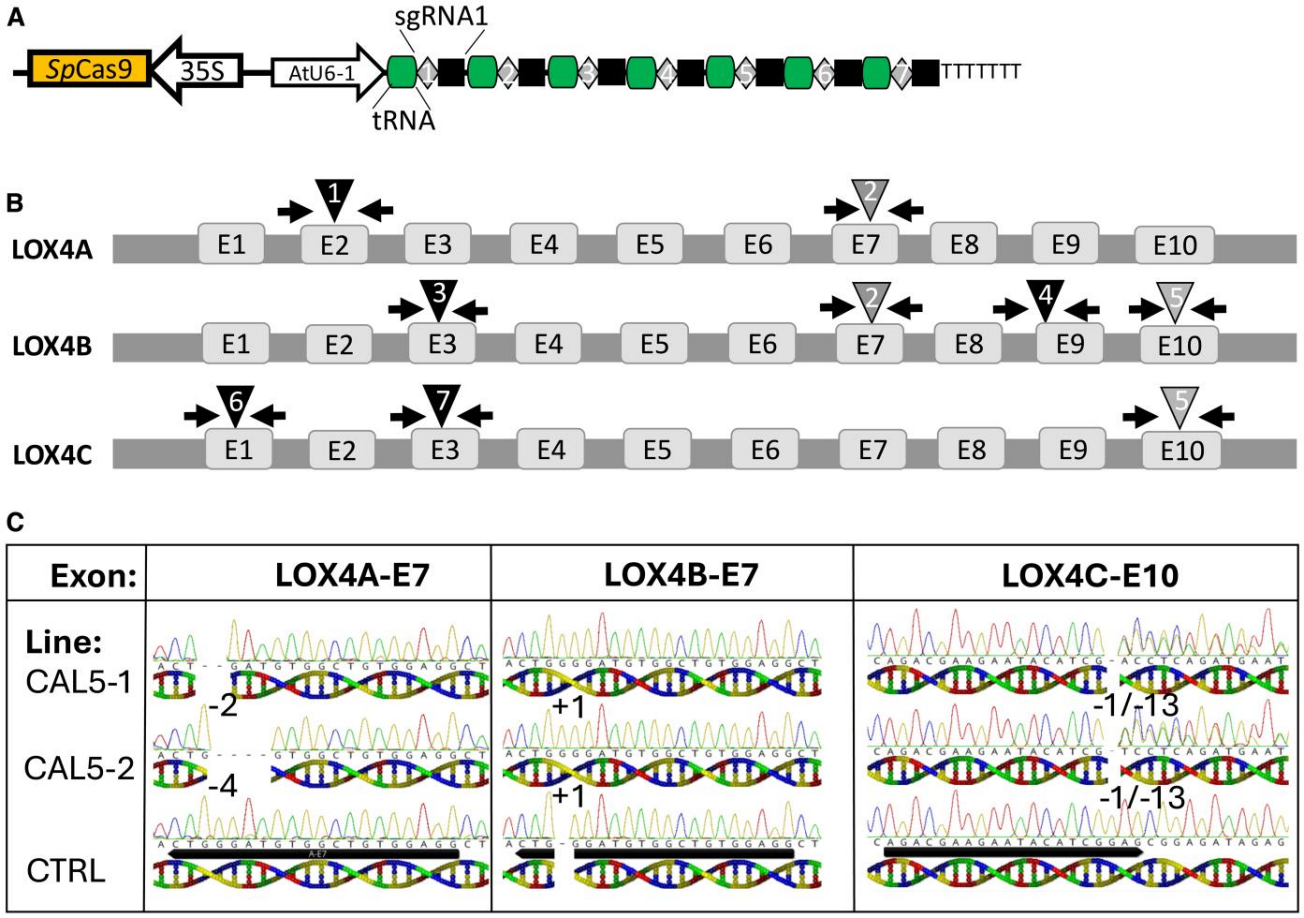
CRISPR-Cas9 construct design, location of guide targets in the 3 *AcLOX4a-c* genes and sanger sequencing analysis of selected transgenic CRISPR lines. **A)** Design of the construct to express the *SpCas9* and single guide RNAs (sgRNA). The tRNAs are indicated in green and below the schematic, the sgRNAs in gray/black. **B)** Location of the 7 guide targets (triangles 1–7) in *AcLOX4a*, *b* and *c* exons 1–10 (E1–10) and primers used for sequencing (black arrows). **C)** Sanger sequencing of insertions and deletions (indels) in lines CAL5-1 and 5-2 in the *AcLOX4a–c* genes. Deletions: −1/−2/−4/−13 and insertions: +1 (in base pairs) compared with control (CTRL)/wildtype. The guide targets are underlined in black under the CTRL track.

To directly demonstrate the importance of *AcLOX4a–c* genes to C6-aldehyde production in fruit and possibly leaves in kiwifruit, a CRISPR knockout strategy was employed, targeting all 3 LOX genes at the ALD locus. Seven guides (E1–7) were designed (Fig. 7A) that targeted exons 1–3 or 7, 9, and 10 in the *AcLOX4a*, *b* and/or *c* genes (Fig. 7B). A comprehensive sequencing strategy was employed to confirm that genome edits were successfully obtained for each of the 9 possible target sites (Supplementary Table S11; Fig. 7C). From the analysis, 5 out of 9 CAL (CRISPR at the Aldehyde Locus) lines showed evidence of genome editing, while 4 lines showed either only wildtype sequences, or were inconclusive (Supplementary Table S11, A and B). Line CAL3 was partially mutated in *AcLOX4b* and *c*, line CAL4 was partially mutated in *AcLOX4a* (63%) and *AcLOC4b* (9%), and CAL9 was partially mutated in *AcLOX4b* only (10%). Lines CAL5-1 and 5-2 showed indels in all 3 target genes, at 94%/98%/77% and 98%/99%/66% efficiency, respectively, to the corresponding genes *AcLOX4a–c* (Fig. 7C).

### Analysis of aldehyde production in LOX CRISPR-edited kiwifruit lines

**Figure 8.**
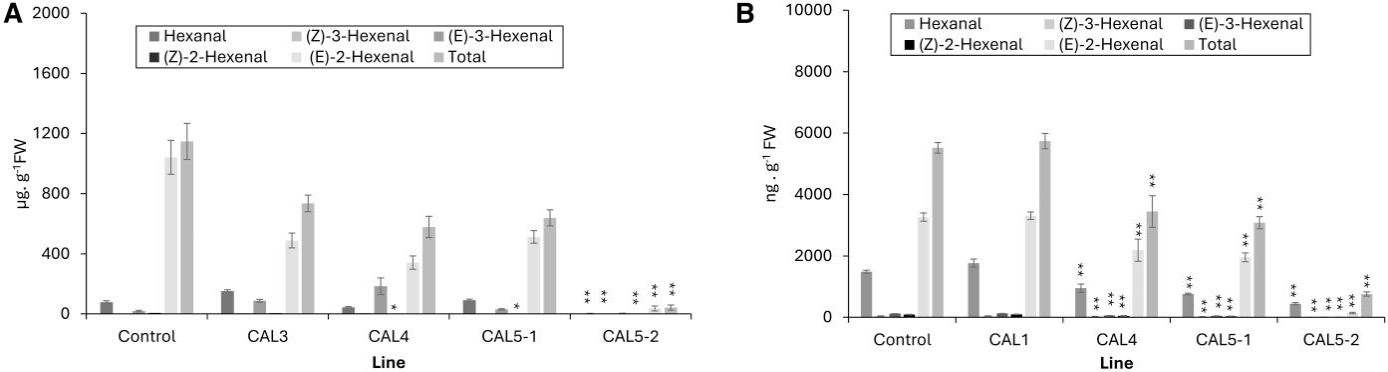
C6-aldehyde concentrations in CRISPR-edited kiwifruit lines. Leaves **A)** and mature fruit **B)** of *AcLOX4a–c* CRISPR-edited and control lines of kiwifruit analyzed by SPME GC-MS. In **B)** line CAL3 was excluded, as only 1 fruit was obtained, and line CAL1 was included as a substitute. Data are mean ± SE from 3 leaf and 5 fruit biological replicates. * Tukey's HSD test *P* < 0.05, **: *P* < 0.01 significantly different from control for indicated VOC values tested (Line CAL4, 5-1, 5-2). Lines CAL3 and CAL1 were not statistically different from control (*P* > 0.05) for all 6 VOC values in leaves and fruit respectively. FW, fresh weight.

Volatiles were initially assessed in the fully-expanded leaves of tissue-cultured LOX CRISPR- edited plants after rooting and transfer to soil in the containment glasshouse. Three edited lines confirmed by sequencing (CAL4, 5-1, 5-2), showed a significant reduction in concentrations of (*Z*)-2-hexenal (Fig. 8A; Supplementary Table S12A) and line CAL5-2 showed significant reduction in all of the most abundant C6-aldehydes (Fig. 8A; Supplementary Table S12A). Lines CAL3, CAL4 and CAL5-1 showed 36% to 49% reduction in total C6-volatiles, however, line CAL5-2 stood out and showed a > 95% reduction in leaf C6-aldehydes (Supplementary Table S12B).

The gene-edited plants grown in the containment glasshouse were generated in an early flowering mutant of *A. chinensis* var. *chinensis* “Hort16A” and flowers emerged within 1–6 months of transfer from tissue culture into soil. Transgenic lines showed normal growth and development with no changes in leaf size or internode length. Time to fruit maturity was between 170 and 180 days after hand pollination for all lines. Samples from fruit of 4 lines (CAL1, 4, 5-1, 5-2) at maturity were analyzed for VOC production by SPME GC-MS. (Figure 8B; Supplementary Table S13). Results showed that CAL4, 5-1 and 5-2 all released significantly reduced concentrations of C6-aldehydes, while CAL1 showed no reduction. CAL4 and 5-1 showed ∼50% reduction in major and minor aldehydes, as well as total aldehydes. The best line, CAL5-2 showed a 70% reduction in hexanal, an 88% to 98% reductions in hexenal isomers and 86% reduction in total C5/C6-aldehyde/alcohol volatiles (Supplementary Table S13). Other minor aldehydes and alcohols, including (Z)-2-penten-1-ol, 1-hexanol and (*E*,*E*)-2,4-hexadienal, were also significantly reduced (Supplementary Table S13) in CAL5-2. Overall, the patterns of reduction correlated well with the leaf analysis, albeit the absolute levels of individual and total aldehydes in control leaves were over 100-fold higher than in control fruit (>1,000 µg total C6-aldehydes g^−1^ FW in leaves). This difference may reflect the much higher chloroplast content in leaves, the principal site of 13-LOX activity and this matches the chloroplast targeting predictions for AcLOX4a and AcLOX4c (Supplementary Table S5).

### Aldehyde concentrations are important for the sensory aroma of kiwifruit

To investigate the contribution of aldehydes to the sensory properties of kiwifruit, a panel of assessors compared the aroma of control fruit versus fruit from the best LOX CRISPR-edited line CAL5-2, first at harvest and subsequently using ripe fruit. Harvest control “Hort16A” fruit showed a volatile profile dominated by aldehydes (Fig. 2A; Supplementary Table S14; Supplementary Fig. S4) while ripe control and CAL5-2 volatile profiles were dominated by ester and alcohol volatiles (Fig. 2B; Supplementary Table S14; Supplementary Fig. S4). Harvest stage CAL5-2 fruit showed markedly reduced levels in all C6-aldehydes and derived alcohols compared with the control, ranging from 5-fold reduction for hexanal, to 147-fold for (*Z*)-3-hexenal (Supplementary Table S14; Supplementary Fig. S4). Total VOCs in harvest stage control fruit were dominated by aldehydes, representing 84% of total volatiles, which dropped to 45% for CAL5-2 with a 14-fold reduction in total aldehydes compared with control. At the ripe stage, both the control and CAL5-2 fruit VOCs were dominated by the esters methyl- and ethyl butanoate, with the total ester fraction representing more than half of the total volatiles in both lines (Supplementary Table S14). The aldehyde fraction in control ripe fruit dropped to ∼30%, while this represents only 9% in CAL5-2. Some differences were also observed in the ester composition. Methyl and ethyl-hexanoate were 9- and 6-fold reduced in CAL5-2 at the ripe stage, suggesting that the C6-hexanoate moiety may be LOX derived. The ketone 1-penten-3-one was reduced between 2.5- and 5-fold lower in the CAL5-2 versus the control, so this compound may also be LOX derived from the intermediate 1-penten-3-ol (Supplementary Table S14).

For consumer sensory evaluation of the fruit at harvest, 3 tests were used: discriminant analysis, an aroma intensity assessment, and descriptor analysis. For the discriminant analysis, a triangle test was used, where each participant was presented with 3 randomized samples (2 were the same and 1 was different) and the participant was asked to evaluate the aroma of samples by smelling from left to right and to select the “different” sample. Triangle tests were replicated 3 times for each participant. Results are summarized in Table 2; Supplementary Table S15A. For the fruit at harvest, participants were readily able to distinguish the aroma of the transgenic fruit from control fruit (*P* < 0.01). No major differences were reported by participants in the aroma intensity assessment between the control and transgenic fruit (Supplementary Table S15B). Using descriptor analysis, slightly more (9/20) participants reported grassy aroma for the control fruit than for the LOX transgenic fruit (6/20), but this was not statistically significantly different (Supplementary Table S15C).

**Table 2.** Consumer sensory evaluation of control vs LOX CRISPR-edited fruit by triangle test, aroma intensity assessment and descriptor analysis

Test	Fruit	Participants	Reps	Correct/Total	Statistics
Triangle	Harvest	20	3	31/60	*P* < 0.01
Intensity	Harvest	20	3	…	ns
Descriptor	Harvest	20	1	…	ns
Triangle	Ripe	31	3	37/93	ns
Triangle	Ripe	31	2^a^	28/62	*P* < 0.05

For the triangle test, in double-blind random design, 3 fruit were presented in random order with 1 being different (control or LOX CRISPR-edited). Participants were asked to identify the “different” sample. For intensity assessment, participants in double-blind random design were asked to evaluate a control vs CRISPR-edited fruit sample in random order and score the aroma intensity on a scale of 1 (very weak) to 9 (very strong) with the first sample presented being anchored at 5 on the scale. This test was replicated 3 times. For descriptor analysis, participants were asked to describe in double-blind + randomized design, 1 control and 1 transgenic fruit using a controlled vocabulary. Ns: not significant. ^a^Statistically different at *P* < 0.05 when only replicates 1 + 2 were analyzed.

For the ethylene-ripened fruit, analysis of the triangle discriminant test results from 3 replicates initially showed no statistically significant difference between the control and LOX CRISPR-edited line. However, as some sensory fatigue may have occurred (defined as the inability or decreased ability to perceive an odor over prolonged time of exposure), the first 2 replicates were also analyzed separately. Results showed that fruit from the LOX CRISPR-edited line could be distinguished from the control at the *P* < 0.05 level (Supplementary Table S15A—*P* = 0.035) when omitting the third replicate. Fewer participants were progressively able to distinguish between fruit in each subsequent trial replicate (Supplementary Table S15A—right hand side), supporting the idea that sensory fatigue had occurred.

## Discussion

Flavor quality in kiwifruit is determined by the content of sugars, acids, and a diverse set of VOCs such as aldehydes, esters, terpenes, and alcohols derived from a number of substrate pathways (Garcia et al. 2012; Nieuwenhuizen et al. 2015; Zeng et al. 2020; Souleyre et al. 2022). The complexity and multiplicity of independent metabolic pathways contributing to synthesis of flavor-associated chemicals makes quality improvement in flavor profiles a big challenge. To better understand and ultimately improve flavor, this study sought to dissect the contribution of C5/C6-aldehydes to kiwifruit flavor. Using a genetic approach, the ALD locus was identified for hexanal, (*E*)-2 hexenal and penten-3-ol production in kiwifruit which co-located with 3 tandemly arrayed 13-LOX genes (*AcLOX4a–c*) on the top of Chr 28. Of these 3 genes, *AcLOX4a* and *AcLOX4c* can both produce C5/C6-aldehydes when transiently overexpressed *in planta.* The higher expression of *AcLOX4a*, combined with the biochemical characterization suggests it is likely to be the gene contributing most to aldehyde production during fruit development and ripening (Fig. 5) as well as in vegetative tissues (Supplementary Table S9; Fig. 5E). *LOX4b* is a lowly expressed, truncated pseudogene. The results with line CAL4, which is a partial (63%) mutant of *AcLOX4a* and a very weak mutant of *AcLOX4b* (<10%) further support that *AcLOX4a* is the most important gene in the ALD locus, as this line showed about a 50% reduction in fruit aldehydes.

In Arabidopsis and tomato, members of the 13-LOX clade have been shown to be important for jasmonate production e.g. *AtLOX2*-6 in Arabidopsis (Chauvin et al. 2013) and *TomloxD* in tomato (Yan et al. 2013). *AtLOX2* and *TomloxC*, have been shown to be important for C5/C6-volatile production (Chen et al. 2004; Mariutto et al. 2011; Shen et al. 2014; Mochizuki et al. 2016), while *TomloxF* is induced by *Pseudomonas putida* infection (Chen et al. 2004; Mariutto et al. 2011; Shen et al. 2014; Mochizuki et al. 2016), and these genes cluster together with *AcLOX4a–c* from kiwifruit in the same phylogenetic subclade 1 (Fig. 4C). CRISPR gene-edited knockout lines with several frameshift mutations in the *AcLOX4a–c* genes showed up to a 96% reduction in total aldehyde levels in leaves and up to 86% reduction in fruit (highest reduction in line CAL5-2). Lines CAL5-1 and 5-2 had similar levels of edits in all 3 genes, but line CAL5-2 showed the most complete knockout of *AcLOX4a* (98% indel), which suggests that even small amounts of intact mRNA of *AcLOX4a* in line CAL5-1 (<6% of wildtype) can still support around 50% of wildtype levels of C5/C6-aldehydes in both leaf and fruit. In fruit, hexanal was reduced by 70%, while (*E*)-2-hexanal was 88% reduced in line CAL5-2 and to a lesser extent the *AcLOX4a–c* mutations contributed to reduced C5-volatile release such as (*E*)-2-pentenal, 1-pentanol, and penten-1-ol isomers and C6-dienes such as (*E,E*)-2,4-hexadienal (Supplementary Table S13).

Hexanal and (*E*)-2 hexenal are important constituents of kiwifruit flavor (Wang et al. 2011), and have been proposed to represent the green/grassy volatile aroma notes found in abundance in fruit of all kiwifruit species, especially in green-fleshed varieties. Consumer sensory evaluation using a panel of assessors was used to directly test the impact of C6-aldehydes on aroma perception in LOX CRISPR-edited fruit of line CAL5-2, which showed the largest reduction in hexanal and (*E*)-2-hexanal. Participants were able to distinguish the low aldehyde fruit aroma in the LOX gene-edited line from that of wildtype levels in the control when assessing unripe fruit at harvest. However, after ethylene treatment, when ester levels became the dominant volatiles, participants likely suffered from sensory fatigue, and could distinguish transgenic from wildtype aroma only in the first 2 replicates. These experiments were performed using fruit from transgenics with a “Hort16A” background that is a low aldehyde producer (Fig. 3). This genotype was used as it is an early flowering diploid kiwifruit model plant that can be used for rapid transgenic fruit evaluation and can conveniently be re-transformed using hygromycin selection (Varkonyi-Gasic et al. 2019; Wang et al. 2021). It will be interesting to test in future, whether consumers find it easier to detect loss of green/grassy aroma differences in cultivars that have much higher base levels of green/grassy aromas than “Hort16A,” such as “Hayward” or “Zesy002,” or in cultivars that produce lower ester/alcohol levels during the later ripening stages characterized by autocatalytic ethylene production (Atkinson et al. 2011).

From our analysis, leaves produced >100-fold higher levels of C6-aldehydes compared with fruit. This difference could partially be explained by the higher chloroplast content in leaves, the principal site of C6-aldehyde production (Feussner and Wasternack 2002). Green-fleshed kiwifruit cultivars such as “Hayward” and “Bruno” are also associated with green/grassy notes and higher levels of C6-aldehydes in fruit, while many gold-fleshed cultivars such as “Hort16A” are associated with lower levels of C6-aldehydes and less associated green/grassy aromas. In gold-fleshed kiwifruit development, fruit go through a process of de-greening, whereby chloroplasts are broken down into colorless catabolites, thereby revealing the yellow carotenoid pigments (Gambi et al. 2018). However, reduced C6-aldehydes in gold-fleshed fruit cannot be simply attributed to a loss of chloroplasts, as “Zesy002,” a gold-fleshed cultivar is associated with high C6-aldehyde production (Cozzolino et al. 2020).

CRISPR-edited *AcLOX4a–c* lines also revealed the importance of the ALD locus to C6-volatile production in leaves. C6-aldeydes in leaves (also called green leaf volatiles, GLVs) are an important part of the volatile blend that is released upon herbivory by insects and attack by pathogens (Hattula and Granroth 1974; Blée 1998) or after physical wounding. These chemicals can function as signaling compounds between either plants of the same species, of different plant species, or even insects (Scala et al. 2013a). They can also be part of the volatiles released upon pathogen infection. On the one hand C6-volatiles can act as antimicrobial agents, while on the other hand, they may impart susceptibility to bacterial infection by affecting the phytohormone balances in plant tissues (Scala et al. 2013a, 2013b), and perturbations can affect substrate and defense signaling crosstalk (Halitschke et al. 2004). It will be interesting to study whether any correlation exists between the C6-volatile levels in fruit and leaves in kiwifruit in wider germplasm, as they appear to be controlled by the same ALD locus.

The production of C6-aldehydes associated with green/grassy notes has been correlated with LOX pathway gene expression in many species. The importance of these green/grassy notes is likely to be especially important in green fruit consumed as vegetables/in savory dishes such as cucumber and olive but has only been directly linked to flavor perception in tomato fruit, while in other cases such as legumes they may act as “aroma modifiers” depending on concentrations and combinations in aroma blends (Trindler et al. 2022). Our results in kiwifruit, a sweet fruit, show that aldehydes are important contributors to flavor intensity, even where esters are the dominant aroma compounds. Consumer preference is likely to be driven by the balance between volatile classes, with fruit showing low flavor intensity or unbalanced profiles, being less attractive and bland tasting. From an evolutionary perspective, studies have suggested that aliphatic esters, mainly responsible for the fruity notes, may have evolved as a predictor of sugar content/reward (“honest signal”) for frugivores as the alcohol moieties of esters are often derived from cell wall degradation/softening (methanol) and sugar fermentation (ethanol) (Nevo et al. 2022). We propose that elevated green/grassy aromas are part of the unripe signals that declines during fruit development in kiwifruit, but still are an important contributor to characteristic kiwifruit fruit flavor. Understanding the genetics for aldehyde and ester production in kiwifruit should enable breeders to produce novel kiwifruit cultivars more rapidly, with the most desirable aroma compositions.

## Materials and methods

### Volatile analysis of fruit at harvest and when eating ripe

Kiwifruit tissue from the cultivars and genotypes described in Fig. 2 was collected at harvest and when eating ripe (<0.7 > 0.2 kgF firmness). Samples were pulped and volatile analyses were conducted by either dynamic headspace sampling combined with direct thermal desorption (DTD) GC-MS, SPME GC-MS; or by SE GC-MS as indicated in Supplementary Table S1.

DTD GC-MS analysis used the method described by Wang et al. (2011), with minor modifications. Volatile were trapped for 20 min, in a DTD tube (ATAS GL International, Eindhoven, The Netherlands) packed with 80 mg of 60 to 80 mesh Chromosorb 105 absorbent (Shimadzu Co. Ltd, Kyoto, Japan), using purified air at a flow rate of 25 mL·min^−1^. Headspace volatiles were desorbed directly from the DTD tubes, using an Optic 3 thermal desorption system (ATAS GL), onto a 30 m × 0.25 mm × 0.25 μm film thickness DB-Wax (J&W Scientific, Folsom, CA, USA) capillary column in a HP6890 gas chromatograph (GC) (Agilent Technologies, Santa Clara, CA, USA). Peaks were identified by time-of-flight mass spectrometry (TOF-MS, Leco Pegasus III, St Joseph, MI, USA). Thermal desorption from the DTD tubes was at 175 °C for 100 s. During desorption the volatiles were cryo-focused on the GC column at −110 °C using low pressure flow of liquid nitrogen. The focused volatiles were then flushed down the column by heating at 175 °C using 2 mL·min^−1^ of helium. The GC oven temperature program was: 35 °C for 2 min, 3 °C·min^−1^ to 60 °C, 5 °C·min^−1^ to 100 °C, 8 °C·min^−1^ to 170 °C, 10 °C·min^−1^ to 200 °C, and hold for 13 min. Temperature of transfer line to MS and the ion source was at 200 °C. Electron impact energy was 70 eV, the acquisition rate was 20 spectra·s^−1^, and the mass range was 35 to 320 (*m*/*z*). The amount of each chemical was calculated as ng·h^−1^·g^−1^ FW with the use of an external standard containing the major terpenes. Only volatiles representing over 1% of the total content are shown.

SPME GC-MS analysis was performed using a method modified from Zeng et al. (2020). Volatiles were collected at 45 °C for 15 min after addition of 0.4 g·g^−1^ of NaCl. Samples were stirred with a Gerstel agitator at 250 rpm controlled by a multipurpose sampler (Gerstel). A SPME fiber (1 cm) coated with 50/30 µm Carboxen/DVB/PDMS (Stableflex) was used for the volatile collection. Separation was performed using a 30 m × 0.25 mm internal diameter × 0.25 μm film thickness DB-WAX-UI (Agilent) capillary GC column in a HP7890B GC system (Agilent) coupled to a Pegasus BT TOF mass spectrometer (Leco). GC temperature programs were 45 °C for 1 min, 6 °C·min^−1^ to 80 °C, then 10 °C·min^−1^ to 160 °C, and then 20 °C·min^−1^ to 240 °C (hold 8 min). Temperature of the transfer line was 240 °C. Mass spectra (*m*/*z* 35 to 350) were collected at an acquisition rate of 30 spectra·s^−1^. Volatiles were identified by comparison with NIST V2.3, 2017 library and confirmed by comparison of retention indices with those of authentic standards and literature values. Sample peak areas were converted into ng·g^−1^ by comparison with the internal standard cyclohexanone (2.049 μg per sample) or hexadecane standard added in each sample.

SE GC-MS analysis was performed using a modified method from Matich et al. (2003). Pulp samples were directly extracted with 10 mL and then 5 mL of pentane:ether (1:1). Extracts were dried by passing them through a small column of anhydrous MgSO_4_, then concentrated to ∼50 µL under a gentle stream of nitrogen. Chromatographic separations were carried out in SP10-Wax (Supelco, Belefonte) or ZBWax capillary columns (Phenomenex, Torrance, California, USA) as described in Matich et al. (2003). Compounds were quantified by comparing their total ion current intensity with that of ethyl butanoate for esters, hexanol for alcohols, acids, and aldehydes, and α-pinene for terpenes and hydrocarbons (Matich et al. 2003).

### Volatile analysis in the *A. chinensis* mapping population and QTL mapping

The *A. chinensis* mapping population AcMPO was grown in the Plant and Food Research orchard in Te Puke, Bay of Plenty, New Zealand, and open pollinated. The female parent (MpM) originated from seed from Henan province, Central China. The male parent (MpM) came from a seed accession from Guangxi province, South China (Fraser et al. 2009). Eating ripe fruit from 117 female individuals was phenotyped in triplicate (3 biological replicates) for VOCs using 2 analytical methods: (ⅰ) dynamic headspace sampling of pulped fruit (1.5 g) followed by direct thermal desorption (HS-DTD) and (ⅱ) SE by pentane:ether (1:1) extraction of 10 g pulped eating ripe fruit (ripened at room temperature in air) followed by solvent injection and GC-MS analysis as described previously (Souleyre et al. 2022). For each vine, the 3 replicates each consisted of 10 eating-ripe fruit (firmness ∼0.5 kgF) that were mixed together.

The resulting data files were analyzed for normal distribution (R-functions Hist and Qqnorm) and were transformed by adding the value 1 to all data if any values were 0 or <1 (data + 1) or log10 transformed of (log10) when data showed a lognormal distribution. ANOVAs were carried out in GenStat (VSN International Ltd.), and only associations with Fprob <0.001 were reported. For some genotypes, fruit for headspace analysis was collected on multiple harvest dates. Both the 1st harvest (1st) and the mean values of all harvests for each genotype (all) were analyzed separately.

### Phylogenetic analysis

Sequence alignments were conducted using Geneious Prime software (v2022.0.1—www.geneious.com) using the “Muscle Alignment” tool with default parameters and trees were generated using the “Geneious Tree builder” tool with the Jukes-Cantor genetic distance model and UPGMA build method and 1,000 bootstraps.

### Volatile analysis during fruit development and ripening of 4 kiwifruit genotypes

Vines were grown at the PFR orchard in Kerikeri, New Zealand and fruit harvested at monthly intervals starting from 30 d after full bloom (DAFB), until commercial harvest i.e. with soluble solids content (SSC) >6.3° Brix (Supplementary Fig. S5). Commercial harvest (H0) was 180 DAFB for “Hort16A” and MpM, 194 DAFB for “Zesy002” and 223 DAFB “Hayward”. Fruit was treated for 20 h with 100 ppm ethylene to synchronize ripening and sampled from 0 to 11 d (H0–H11) after treatment, depending on the genotype. Parameters such as fruit weight, ethylene production, SSC, firmness and ethylene production were measured (Supplementary Fig. S5). Fruit firmness was measured using the Instron Universal Testing Machine (Model 4301; Instron, Inc. Canton, MS, USA). Soluble solids concentration (SSC) was measured using a digital refractometer (Atago, model PAL-1; Japan). Endogenous climacteric ethylene production of individual fruit was monitored by placing fruit in 529 mL respiration containers for 1 h. Samples (1 mL) were then withdrawn from the container headspace and ethylene measured by flame ionization chromatography (PU 4500 Chromatograph; Phillips, UK) as described in (Johnston et al. 2009). Volatiles were sampled by SPME GC-MS as described in M&M, paragraph 3. For “Hort16A” fruit development (30 d to 180 d), fruit were sampled by DTD GC-MS as described in M&M, paragraph 2.

### RT-qPCR gene expression analysis

RNA from leaf, developing fruit and ripe fruit was isolated using the Spectrum Plant Total RNA Kit (Sigma-Aldrich). An aliquot of 1 µg total RNA was reverse transcribed using the QuantiTect Reverse Transcription Kit (Qiagen). Quantitative real-time PCR (RT-qPCR) was performed with the FastStart DNA Master SYBR Green I mix (Roche Diagnostics) using the LightCycler 1.5 instrument and the LightCycler Software version 4 (Roche Diagnostics). Ampliﬁcation was carried out using a 50× dilution of the cDNA template, with an initial denaturing step at 95 °C for 5 min, then 40 cycles of 95 °C for 5 s, 60 °C for 5 s, and 72 °C for 15 s. A nontemplate control was included in each run. Oligonucleotide primers (Supplementary Table S8) were designed to produce ampliﬁcation products of 80–120 nucleotides in length. The speciﬁcity of primer pairs was conﬁrmed by melting curve analysis of PCR products and the efficiency of each primer pair was determined by serial dilution. The expression was normalized to the geometrical mean of the kiwifruit EF1α (Nieuwenhuizen et al. 2021) and the ubiquitin (UBI9) reference gene after evaluating 4 reference genes (Supplementary Table S8), and presented as a mean expression ± standard error (SE) of 3 biological replicates each based on 4 technical replicates.

### Heterologous expression of LOX genes

For transient *in planta* expression, the complete open reading frames of all predicted 13-LOX genes and alleles were PCR amplified and cloned into the vector pHEX2 (Hellens et al. 2005) by Gateway cloning (Invitrogen, USA) using primers listed in Supplementary Table S8 and sequence verified. All genes/alleles were amplified from ripe MpM fruit cDNA, except alleles *AcLOX4a* (4a-D) and *AcLOX4c* (4c-D) that were amplified from expanding leaf cDNA from MpD. pHEX2 carries the CaMV 35S promoter and octopine synthase terminator (Hellens et al. 2005). *Agrobacterium tumefaciens* GV3101 harboring the pHEX2-LOX constructs (OD_600_ = 0.5) were infiltrated into the 4th and 5th true leaf of 3 to 4 week old *N. benthamiana* plants (Hellens et al. 2005). After 7 d, leaves were harvested in liquid nitrogen and ground to a fine powder using a mortar and pestle. VOCs from 0.5 to 2 g of frozen *N. benthamiana* leaf powder were analyzed by SPME GC-MS as described in M&M, paragraph 3. All the experiments were conducted with at least 3 biological replicates.

For heterologous expression in *E. coli*, the complete open reading frames of AcLOX4a-D1 and AcLOX4c-D1 minus the predicted chloroplast targeting peptide were PCR amplified and cloned by In-Fusion cloning (Takara Bio, Japan), into the EcoRV/BamHI restriction sites of the expression vector pMAL-c6T (New England Biolabs, USA). Primers are listed in Supplementary Table S8. Recombinant HIS6-MBP fusions were then overexpressed by autoinduction in *E*. *coli* BL21 (DE3) Codon Plus RIL cells (Stratagene, La Jolla, CA, USA) and purified to near homogeneity using nickel affinity purification (HIS-TRAP chelating HP, Cytiva, USA) as described previously (Nieuwenhuizen et al. 2009) by elution with a continuous 0 to 500 mm imidazole gradient. Peak fractions were analysed by SDS-PAGE for purity (Supplementary Fig. S3) and desalted into 30 mm Tris-Cl, pH 7.5 buffer, flash frozen in liquid nitrogen and stored at −80 °C until use. Biochemical analysis was done in air saturated 30 mm Tris-Cl, pH 7.5 and substrates were prepared freshly and assayed as previously described (Maynard et al. 2021). α-Linolenic acid (≥99%, L2376) and linoleic acid (≥99%, L1376) were purchased from Sigma (Darmstadt, Germany). Using the spectrophotometric approach, LOX-catalysed epoxide formation was monitored at 234 nm (ε = 23,000 L·mol^−1^·cm^−1^) for 3 min at 10 s intervals and the maximum rate of product formation was determined for kinetics calculations.

### Generation of CRISPR-Cas9 constructs to knockout *AcLOX4a–c*

*AcLOX4a–c* target sites were designed using Geneious software (Geneious Prime software v2022.0.1—www.geneious.com) using the kiwifruit genome sequence (Red5v1.69) as off-target database to identify and avoid any potential off-targets and further verified by BLASTn searching (Supplementary Table S11C). CRISPR-Cas9 knockout constructs were generated in the vector pDE-KRS-HYGR. This vector was generated by replacing the nos-nptII cassette from pDE-KRS (Varkonyi-Gasic et al. 2019) with a 35S-promoter-hygromycin B (hpt)-polyA cassette as described in (Akagi et al. 2019). The 7 pre-tRNA-guides followed by a polyT terminator cassette were generated by Golden Gate cloning using *Bsa*I and cloned into pENTR-AtU6.26 as described in (Zhou et al. 2018). The resulting plasmid was transferred by Gateway LR cloning (Invitrogen) into pDE-KRS-HYGR to generate the final plasmid which was transformed into *A. tumefaciens* strain EHA105 by electroporation.

### Plant transformation and growth

*A. tumefaciens* mediated transformation of *A. chinensis* var. *chinensis* “Hort16A” U6-CEN4#18 (Varkonyi-Gasic et al. 2019) was performed as previously described (Wang et al. 2007). Brieﬂy, leaf strips excised from in vitro*-*grown shoots were co-cultivated with *Agrobacterium* suspension culture and transferred to regeneration and selection medium containing 10 mg·L^−1^ hygromycin B. Individual calli were excised from the leaf strips for further selection and bud induction, and adventitious buds regenerated from the calli were excised and transferred to shoot elongation medium. When shoots had grown to 1 to 2 cm in height, they were transplanted onto rooting medium. Rooted transgenic plants were then potted and grown in a containment greenhouse at ambient conditions (temperature: min 18 °C/max 30 °C; night/day: 14 h/10 h light/dark in summer, with gradual day length shortening to 12 h/12 h light/dark at the beginning of autumn). For clonal propagation, leaf tissue was surface-sterilized and allowed to regenerate in tissue culture and develop roots and subsequently grown as described above. For CRISPR mutant testing, for each line, a small leaf sample was used for DNA extraction (NucleoSpin Plant II, Mini kit, Macherey-Nagel, Germany). PCR amplicons were designed (Supplementary Table S8 for primers) spanning the guide targets and tested by Sanger sequencing, followed by TIDE deconvolution of the electropherograms (Brinkman and van Steensel 2019). For line CAL4, further sequencing was done by Nanopore MinION (Oxford Nanopore, UK) amplicon sequencing (Supplementary Table S8 for primers), to verify the *AcLOX4a* mutation rate in this line (Supplementary Table S11B).

### Volatile measurements in transgenic leaves and fruit

Rooted transgenic plantlets were transferred from tissue culture to soil in pots and grown under glasshouse conditions with supplemental lighting at 21 °C under 16 h daylength conditions. Flowers were hand-pollinated with diploid male pollen. At least 5 fruit per line were analyzed for fruit volatiles and at least 3 leaves for leaf volatiles. Fruit and leaf tissue were ground under liquid nitrogen and 1 g tissue was mixed with 0.4 g NaCl and measured by SPME GC-MS as described in M&M, paragraph 3.

### Consumer sensory evaluation of LOX CRISPR-edited fruit

All tests were conducted under controlled conditions. Care was taken to ensure that each product sample was prepared and presented in a standardized and consistent manner to ensure that no sources of bias were introduced that could have a negative impact on the end test result. The triangle test was principally used to determine whether a sensory difference existed between 2 fruit: a transgenic fruit from the LOX CRISPR-edited line CAL5-2 vs a control fruit (parental line). Each untrained participant on the panel was presented with 3 randomized samples (2 were the same and 1 was different) and asked to evaluate the samples from left to right, and select the different sample, even if they thought there was no clear difference between the samples.

For the harvest samples, fruit was prepared by using a 1 g cube of flesh from freshly harvested fruit (170 DAFB), which was pulped just before each assessment using a garlic press. The resulting mashed pulp was placed inside a 20 mL plastic cup and sealed with a lid to let aroma volatiles accumulate for 2 min. Each sample was then presented in 3 identical containers covered with aluminum foil to prevent any visual identification, following a balanced experimental test design. Each sample was evaluated following a standardized smelling protocol whereby the lid was lifted from the cup by the participant (*n* = 20) and the sample was smelled immediately, and the lid was closed. After a brief cleansing/neutralization by smelling the wrist between samples, the second and third replicate test were assessed in the same way and the “different” sample was recorded on a paper ballot. Three tests were conducted per participant for a total of 60 tests. The number of correct answers/judgements were counted and compared against statistical tables based on the 1-tailed binomial test as described (Sinkinson 2017) to verify whether it could be concluded that a statistically significant difference existed between the 2 samples.

To record the aroma intensity, the participant was presented with a control and transgenic pulped fruit sample in random order, prepared as above. The participant was then asked to score the intensity of each sample of the pair on a scale from 1 to 9, with 1 being very weak, to 9 representing very intense with the first sample being anchored at a score of 5. This test was repeated twice more (3 replicates). To record any aroma descriptors, after the third replicate of the aroma intensity assessments, participants were asked to describe the smell of the first and second cup by forced choice using a controlled vocabulary (see Supplementary Table S15).

For the ethylene-ripened fruit samples only the triangle test was conducted. At 170 DAFB, harvested fruit was treated overnight (20 h) with 100 ppm ethylene. Samples were assessed by 31 participants; 3 days post the ethylene treatment. Three tests were conducted per participant for a total of 93 tests. Intact fruit cubes (1 cm^3^) of equal weight were used rather than pulp as the ripened fruit was judged to have an overall higher aroma intensity compared with harvest fruit. The same closed and covered containers with lids described for the harvest fruit were used.

### Statistical methods

Correlations were calculated using the Pearson correlation coefficient (R). Statistical differences between multiple groups/treatments were determined using the post hoc Tukey's HSD multiple comparison test after 1 way ANOVA. For the consumer triangle testing, the 1-tailed binomial test was employed (probability: 0.333) (Sinkinson 2017).

### Accession numbers

Sequence data from this article can be found in the GenBank/EMBL data libraries under accession numbers: PQ035133–PQ035138 and Supplementary Tables S6 and S10.

## Supplementary Material

kiaf285_Supplementary_Data

## Data Availability

The data and materials included in the study are available from the corresponding authors upon request.

## Supplementary data

The following materials are available in the online version of this article.

**Supplementary Figure S1.** QTL interval mapping on LG7 for (*E2*)-hexenal and hexanal.

**Supplementary Figure S2.** Alignment of the *AcLOX4a–c* gene models identified in the Red5 genome.

**Supplementary Figure S3.** Kinetics of AcLOX4a-D1 and AcLOX4c-D1 recombinant proteins.

**Supplementary Figure S4.** GC-MS analysis of the most abundant aroma volatiles in fruit of control and CRISPR-Cas9 edited “Hort16A” kiwifruit at harvest and ripe stages.

**Supplementary Figure S5.** Fruit characteristics of 4 kiwifruit cultivars during development and post-harvest.

**Supplementary Table S1.** Volatile classes in fruit at harvest and when eating ripe in kiwifruit cultivars and species.

**Supplementary Table S2.** Volatile classes during fruit development and ripening in 4 kiwifruit cultivars.

**Supplementary Table S3.** Volatile levels in ripe fruit of the AcMPO mapping population by headspace and SE.

**Supplementary Table S4.** QTL mapping of volatile aroma compounds in the AcMPO mapping population.

**Supplementary Table S5.** Microsatellite sequences and location of ALD locus markers.

**Supplementary Table S6.** HPL and LOX genes in the kiwifruit Red5 genome.

**Supplementary Table S7.** Gene candidates within the span of markers associated with the ALD locus.

**Supplementary Table S8.** Primer sequences used in this study.

**Supplementary Table S9.** Pearsons correlation between total aldehydes abundance and LOX expression during fruit development and expression in other tissues.

**Supplementary Table S10.**  *AcLOX4a–c* alleles cloned from the mapping population mother (MpM) and father (MpD).

**Supplementary Table S11.** Tracking of Indels by Decomposition (TIDE) analysis of CRISPR target sites in *AcLOX4a–c*.

**Supplementary Table S12.** Analysis of C6-aldehydes emitted from the leaves of *AcLOX4a–c* CRISPR gene-edited lines.

**Supplementary Table S13.** Analysis of fruit C5/C6-aldehyde and alcohol VOCs in control and CRISPR LOX gene-edited lines.

**Supplementary Table S14.** Volatiles in harvest and ripe control and CAL5-2 fruit used for consumer evaluation.

**Supplementary Table S15.** Consumer sensory results of control versus LOX transgenic fruit.

## Dive Curated Terms

The following phenotypic, genotypic, and functional terms are of significance to the work described in this paper:
