# Supplementary material for: The aldehyde (ALD) locus controls C6-aldehyde production in kiwifruit and affects consumer perception of fruit aroma
Source: Plant Physiol. 2025 Jun 30;198(3):kiaf285. doi: 10.1093/plphys/kiaf285 (PMC12268261; doi:10.1093/plphys/kiaf285)
Supplement: kiaf285_Supplementary_Data [file kiaf285_supplementary_data.zip › Supplementary figures.pdf]

Supplementary Figure S1. QTL interval mapping on LG7 for (*E2*)-hexenal (green) and hexanal (red). Hayley-Knot regression was used based on 1000 permutations. The 1.5 LOD significance interval is presented using the Mapchart software (Voorrips 2002). Both (*E2*)-hexenol and 1-penten-3-ol were not significant using this analysis. ***: Markers that showed significant QTLs based on the single marker analysis **(Table S3)**.

Reference: Voorrips, R.E., 2002. MapChart: Software for the graphical presentation of linkage maps and QTLs. J. Hered. 93: 77-78.

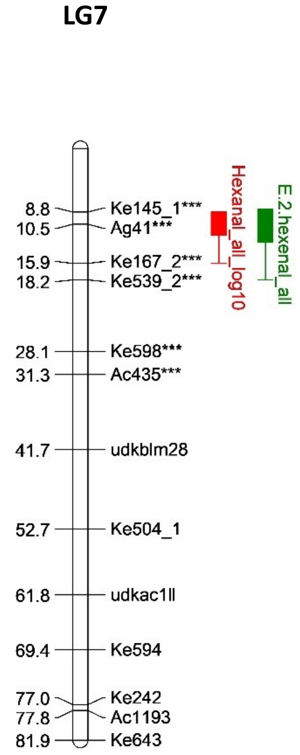

Supplementary Figure S2. Alignment of the *AcLOX4a–c* gene models identified in the Red5 genome. Nucleotide (**A**) and protein (**B**) alignment of the predicted open reading frames of gene models RED5V2.032486.1.1.PC, RED5V2.032487.1.1.PC and RED5V2.032487.1.1.PC (*AcLOX4a*, *-b*, *-c)* respectively based on the Red5 genome sequence version V2; (**C**): Nucleotide identity matrix of the complete alignment; (**D**): Nucleotide identity matrix excluding nucleotides 1-317.

Genome data source: Pilkington, Crowhurst, Hilario, Thomson, Le Leivre, & Brownfield. (2021). *Actinidia chinensis* Red5 genome assembly (version 2) and annotation files (Version 2) [Data set]. Zenodo. https://doi.org/10.5281/zenodo.5717387.

A)

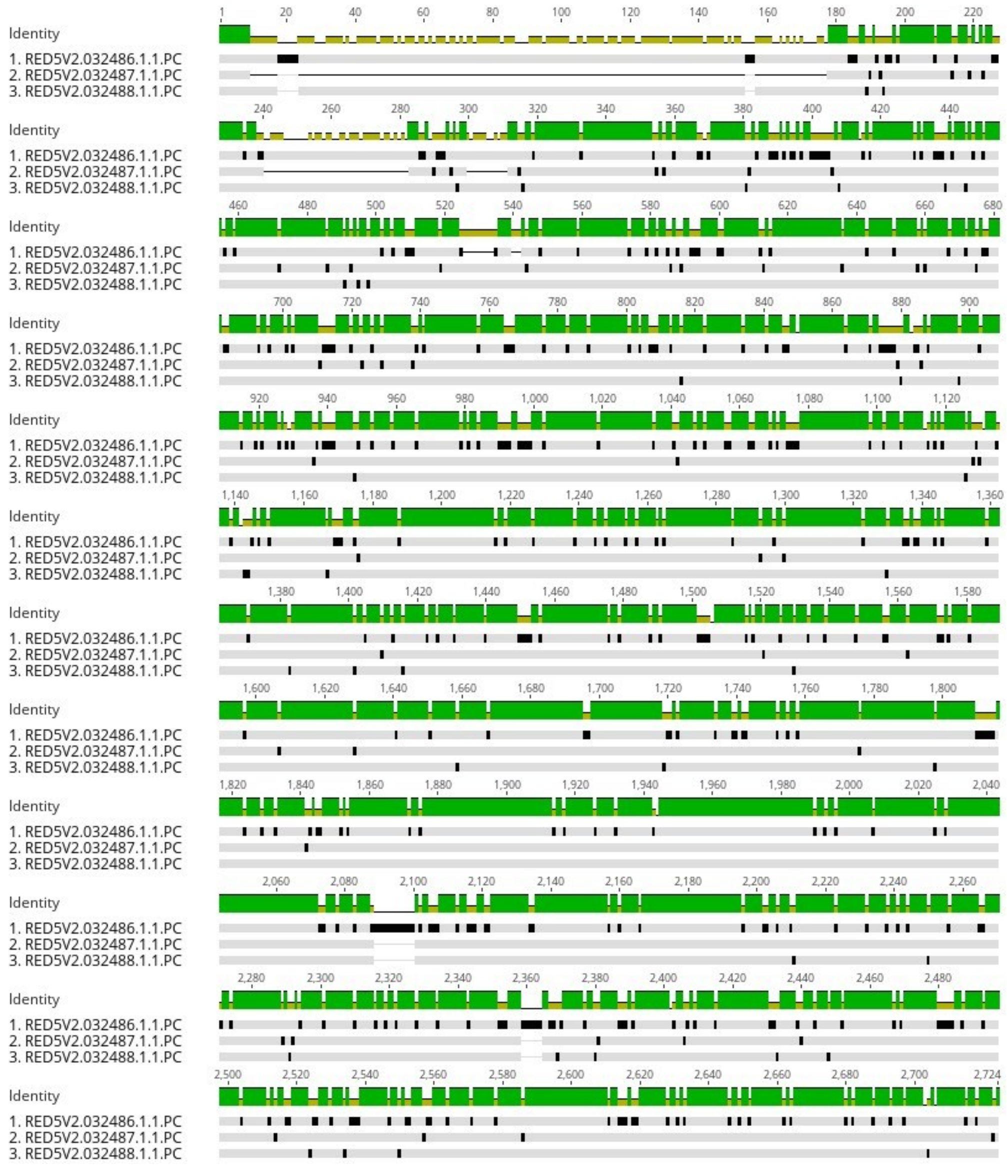

B)

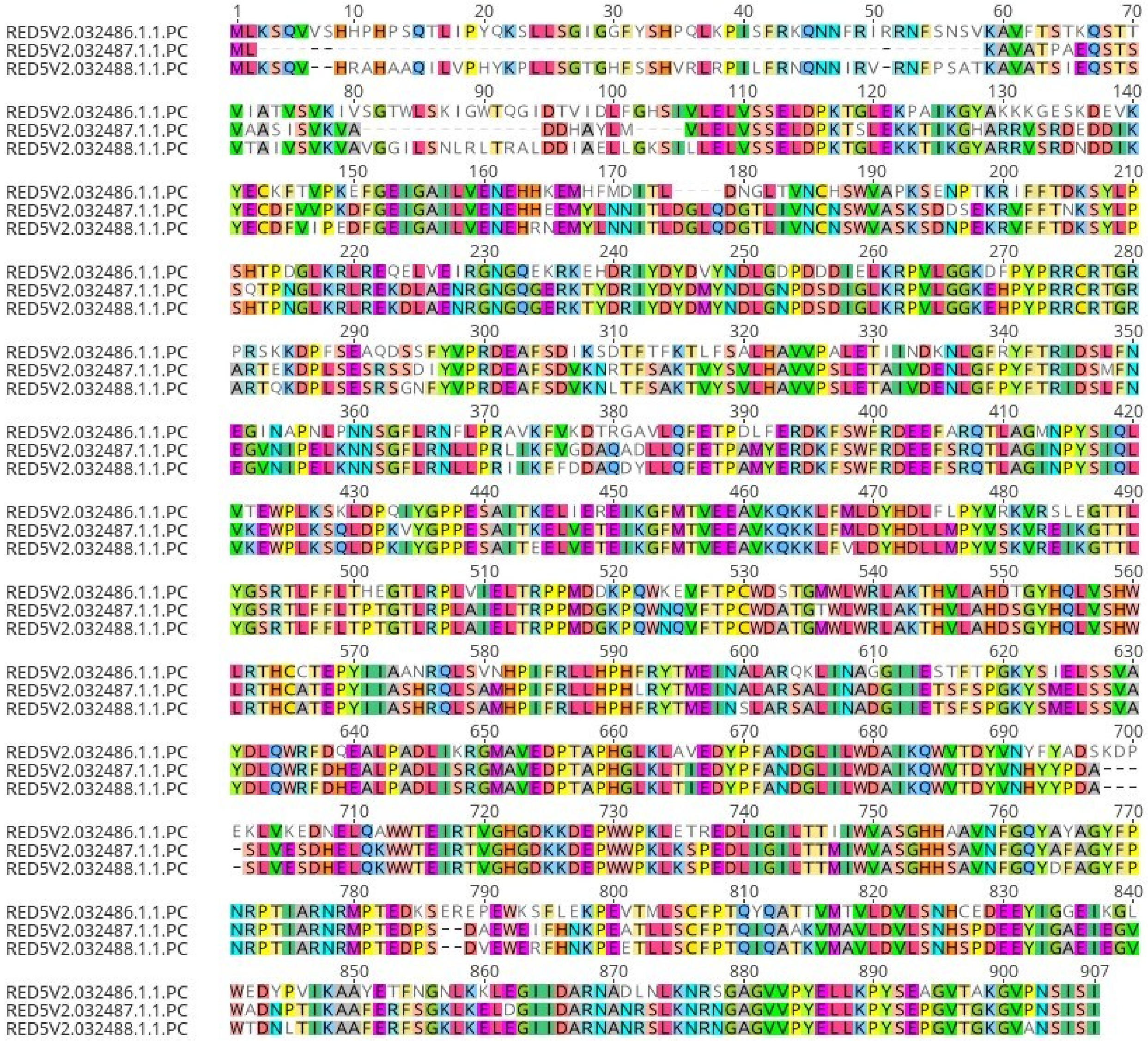

C)

Matrix: % Identity

	RED5V2.03...	RED5V2.03...	RED5V2.03...
RED5V2.032486.1.1.PC		73.972%	79.883%
RED5V2.032487.1.1.PC	73.972%		88.172%
RED5V2.032488.1.1.PC	79.883%	88.172%	

D)

Matrix: % Identity

	RED5V2.03...	RED5V2.03...	RED5V2.03...
RED5V2.032486.1.1.PC		81.0%	81.4%
RED5V2.032487.1.1.PC	81.0%		96.1%
RED5V2.032488.1.1.PC	81.4%	96.1%	

Supplementary Figure S3. Kinetics of AcLOX4a-D1 and AcLOX4c-D1 recombinant proteins. Kinetics were determined using α-linolenic acid (ALA) and linoleic acid (LA) as substrates. **A**. HIS-affinity purified (HIS-Trap HP, Cytiva) and desalted (PD-10, Cytiva) MBP fusion proteins (1 µg/lane) were separated by reducing denaturing SDS-PAGE (4–15% Mini-PROTEAN® TGX Stain-Free™ Protein Gels, Bio-Rad Laboratories). **B**) Kinetic values are derived from nonlinear regression fit using the Michaelis Menten equation with the Origin 2022 software. **C**) The apparent (app) K_m-app and V_{max}-app terms are used to indicate that the 13-LOX enzymes may differ in iron load and display signs of substrate inhibition at high substrate concentrations. For each analysis 3–6 replicates were used per substrate concentration (0–200 µM). Error bars: Standard error.

A)

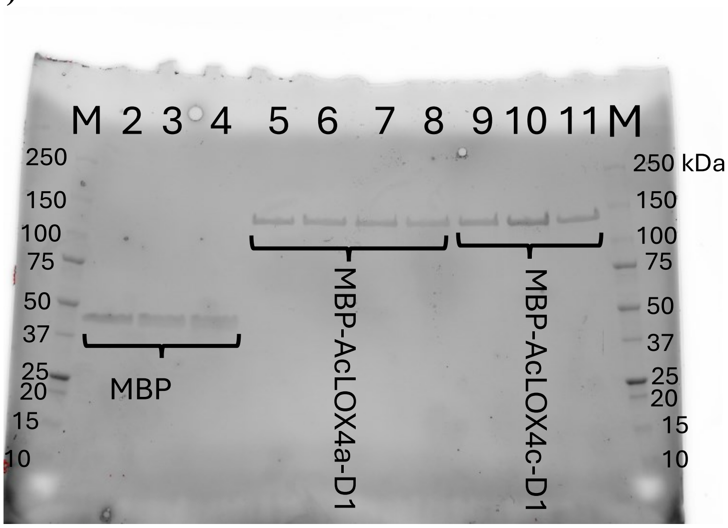

B)

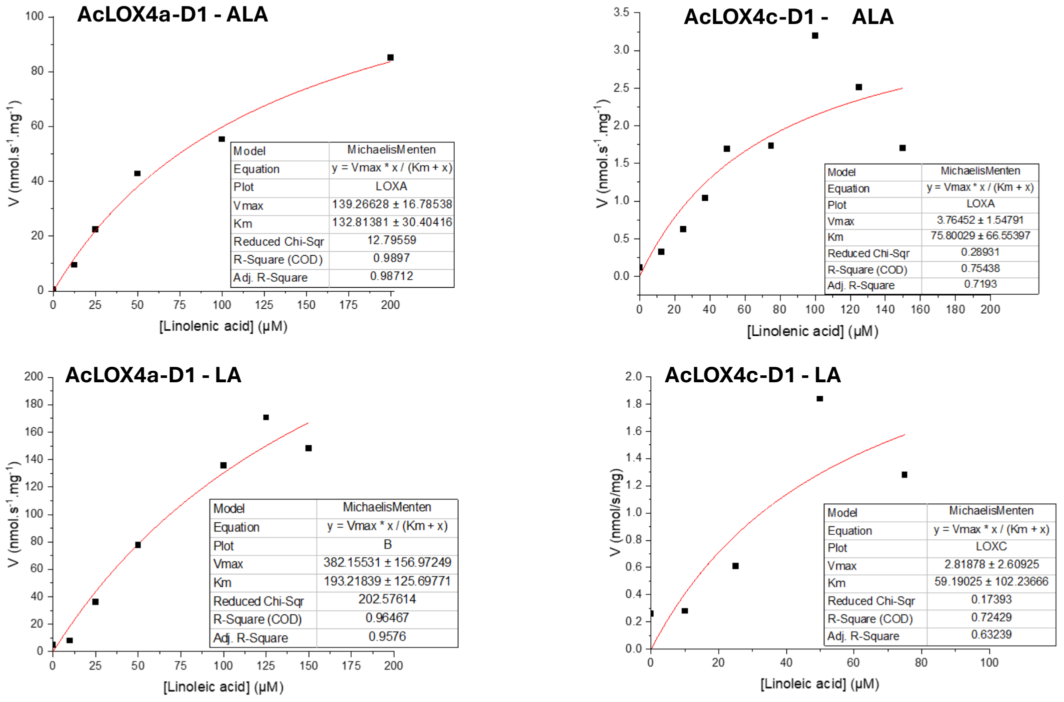
AcLOX4a-D1 - ALA
V (nmol.s-1.mg-1)
[Linolenic acid] (µM)
Model | MichaelisMenten
Equation | y = Vmax * x / (Km + x)
Plot | LOXA
Vmax | 139.26628 ± 16.78538
Km | 132.81381 ± 30.40416
Reduced Chi-Sqr | 12.79559
R-Square (COD) | 0.9897
Adj. R-Square | 0.98712
AcLOX4c-D1 - ALA
V (nmol.s-1.mg-1)
[Linolenic acid] (µM)
Model | MichaelisMenten
Equation | y = Vmax * x / (Km + x)
Plot | LOXA
Vmax | 3.76452 ± 1.54791
Km | 75.80029 ± 66.55397
Reduced Chi-Sqr | 0.28931
R-Square (COD) | 0.75438
Adj. R-Square | 0.7193
AcLOX4a-D1 - LA
V (nmol.s-1.mg-1)
[Linoleic acid] (µM)
Model | MichaelisMenten
Equation | y = Vmax * x / (Km + x)
Plot | B
Vmax | 382.15531 ± 156.97249
Km | 193.21839 ± 125.69771
Reduced Chi-Sqr | 202.57614
R-Square (COD) | 0.96467
Adj. R-Square | 0.9576
AcLOX4c-D1 - LA
V (nmol/s/mg)
[Linoleic acid] (µM)
Model | MichaelisMenten
Equation | y = Vmax * x / (Km + x)
Plot | LOXC
Vmax | 2.81878 ± 2.60925
Km | 59.19025 ± 102.23666
Reduced Chi-Sqr | 0.17393
R-Square (COD) | 0.72429
Adj. R-Square | 0.63239

C)

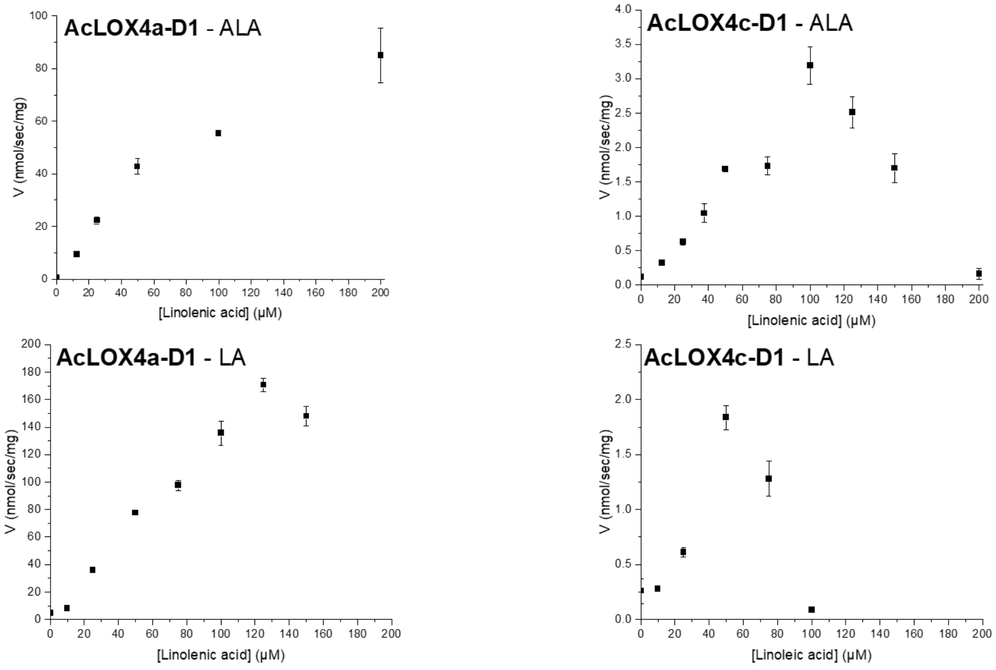
AcLOX4a-D1 - ALA
V (nmol/sec/mg)
[Linolenic acid] (µM)
AcLOX4c-D1 - ALA
V (nmol/sec/mg)
[Linolenic acid] (µM)
AcLOX4a-D1 - LA
V (nmol/sec/mg)
[Linolenic acid] (µM)
AcLOX4c-D1 - LA
V (nmol/sec/mg)
[Linoleic acid] (µM)

Supplementary Figure S4. GC-MS analysis of the most abundant aroma volatiles in fruit of control and CRISPR-Cas9 edited 'Hort16A' kiwifruit at harvest and ripe stages. The eighteen most abundant aroma volatiles found in four treatments: control and CAL5-2 LOX edited fruit at harvest (H) and at the eating ripe (ER) stage (after ethylene ripening). Volatiles are sorted in order of maximum abundance (>50 $ng.g^{-1}$ fresh weight) from left to right. Statistical analysis was performed for each individual compound separately. Different letters ("a" to "d") indicate that statistical differences between the four treatments were found according to Tukey's post hoc HSD multiple comparison test ($p < 0.05$) after one-way ANOVA and based on log10 transformed data. NS: no significant differences between the four treatments were found. At least five biological replicates were used +/- standard error (SE).

All C6-aldehydes, such as hexenal isomers and hexanal and derived alcohols (hexenols/hexanol), are much reduced in the CAL5-2 line, independent of ripening. Esters, such as ethyl-/methyl butanoate and -propanoate, and alcohols, such as ethanol, are ripening/ethylene induced in both genotypes. Esters such as ethyl-/methyl hexanoate are also lower in ripe CAL5-2 compared to control fruit. Ethyl acetate and cyclohexanone (internal standard) show similar abundance, independent of ripening stage and LOX CRISPR knockout status (see also Table S13).

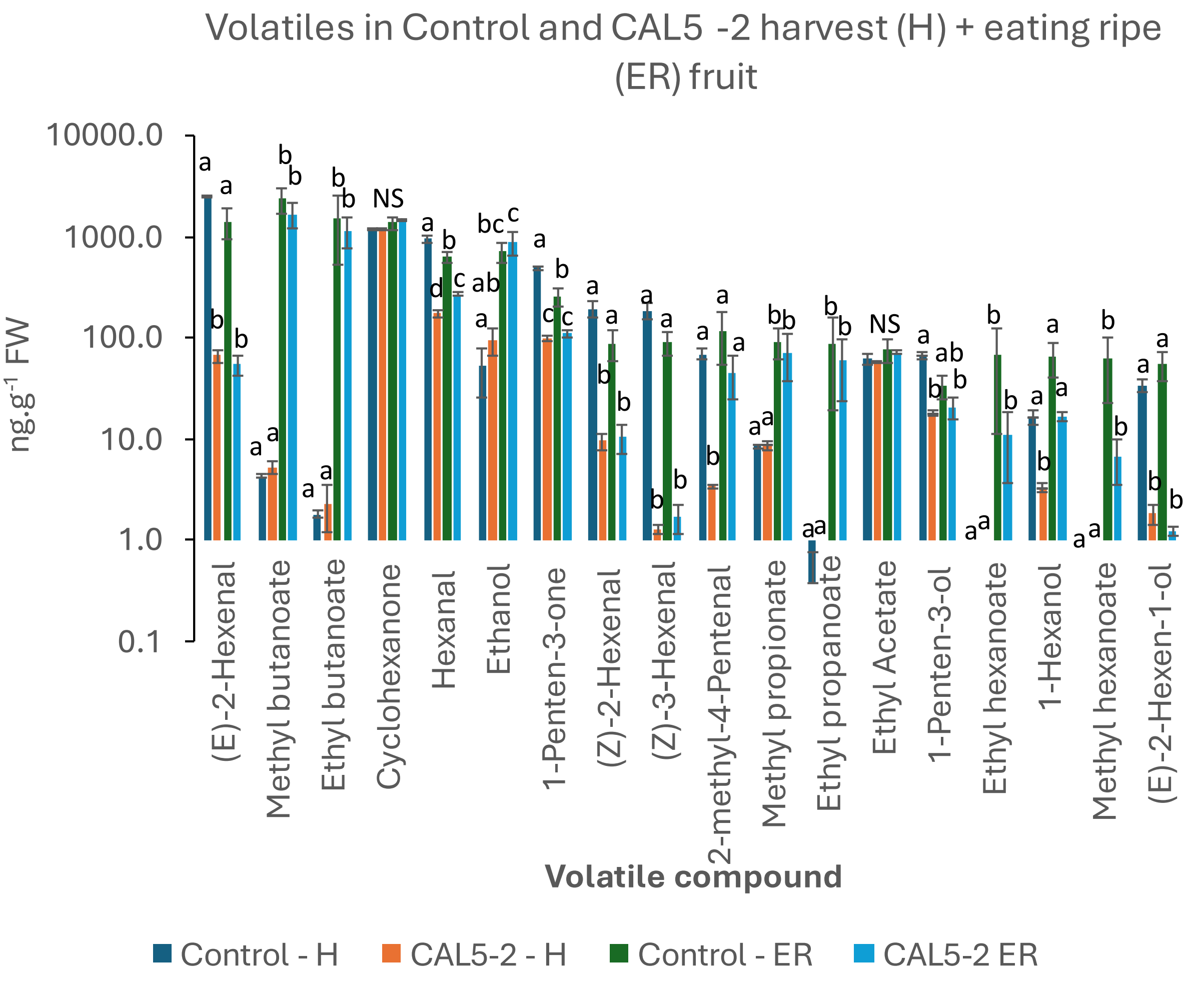

Supplementary Figure S5. Fruit characteristics of four kiwifruit cultivars during development and post-harvest. Fresh weight (**A**), firmness (KgF) (**B**), soluble solids content (SSC in °Brix) (**C**), dry matter (% DM) (**D**), ethylene production **(E)** for *Actinidia* fruit collected monthly (30/60/90/120/150/180 days) and 'H' days after harvest /ethylene induced ripening (H0–H11) of 'Zesy002', MpM , 'Hayward' and 'Hort16A' fruit. A-E ±standard error (n = 3-6 biological replicates).

A)

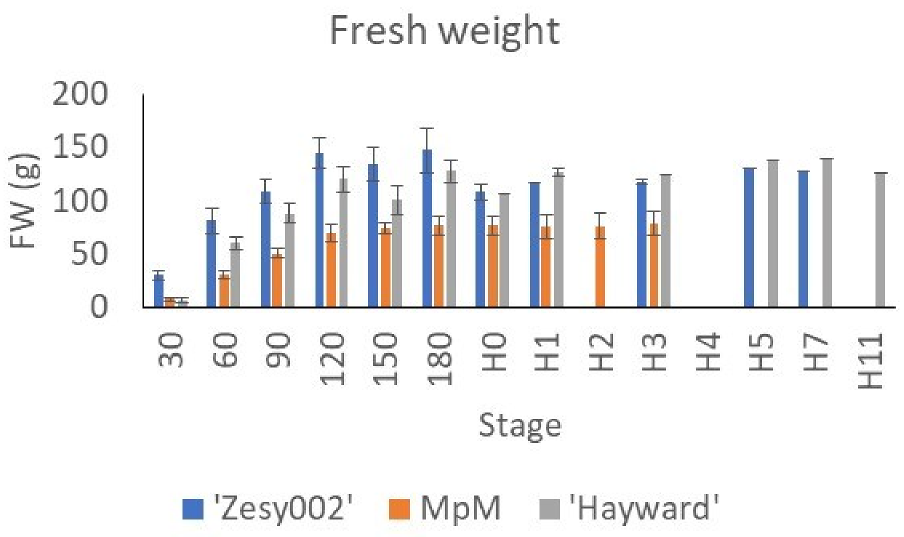

B)

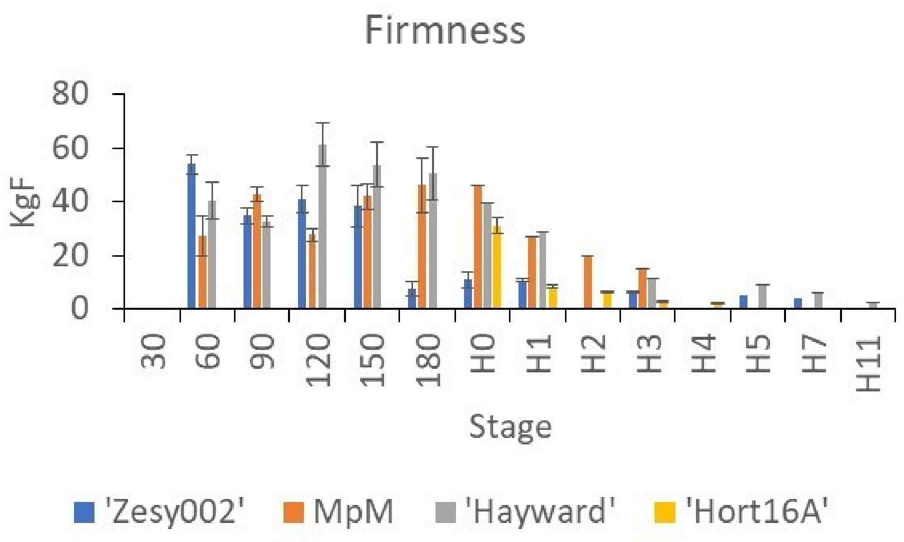

C)

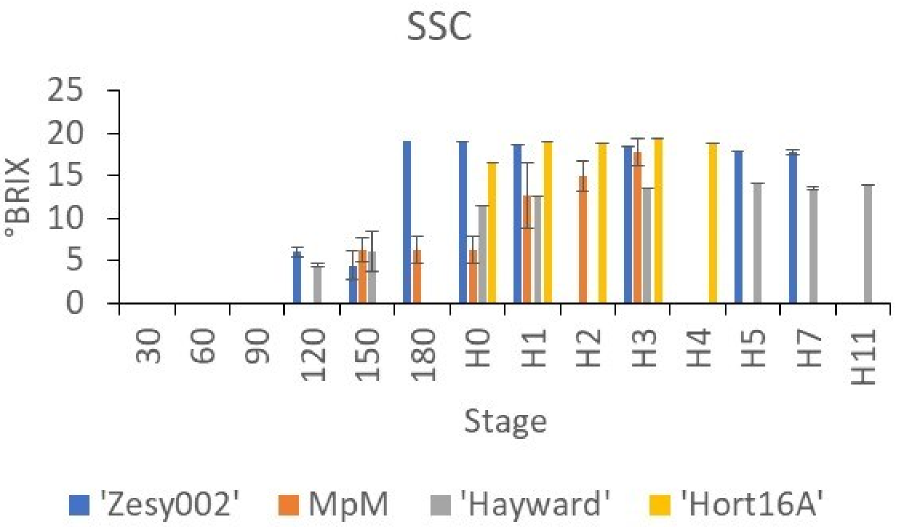
SSC
25
20
15
10
5
0
°BRIX
30
60
90
120
150
180
H0
H1
H2
H3
H4
H5
H7
H11
Stage
'Zesy002'
MpM
'Hayward'
'Hort16A'

D)

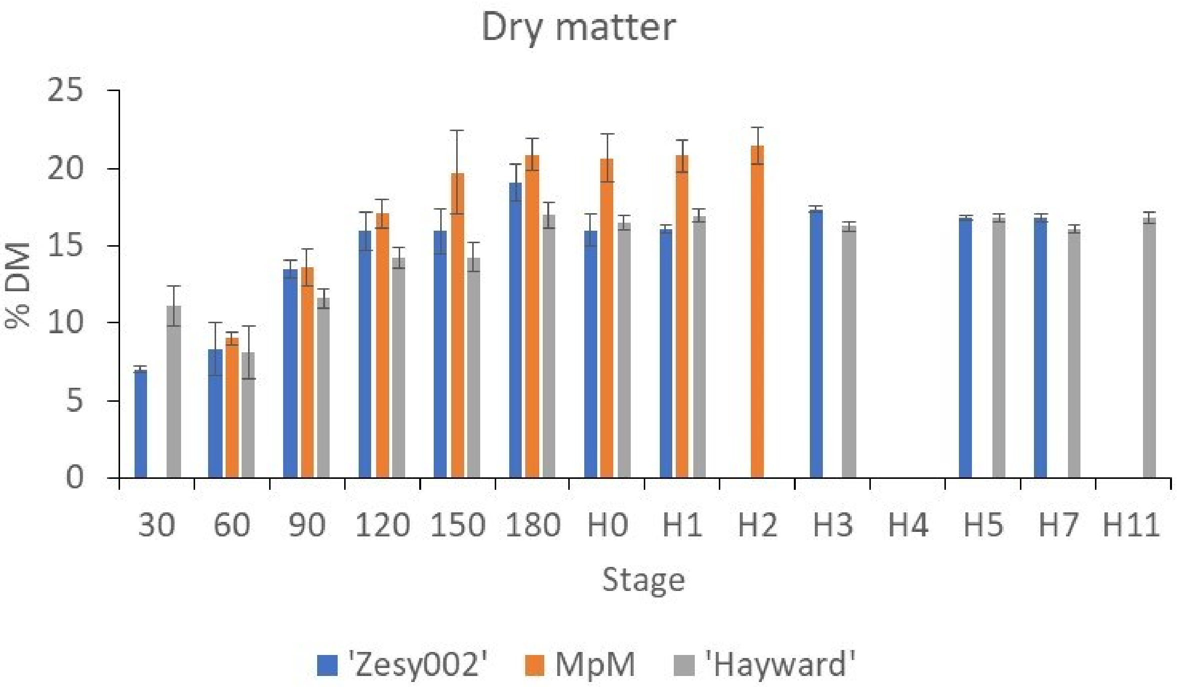
Dry matter
25
20
15
10
5
0
% DM
30
60
90
120
150
180
H0
H1
H2
H3
H4
H5
H7
H11
Stage
'Zesy002'
MpM
'Hayward'

E)

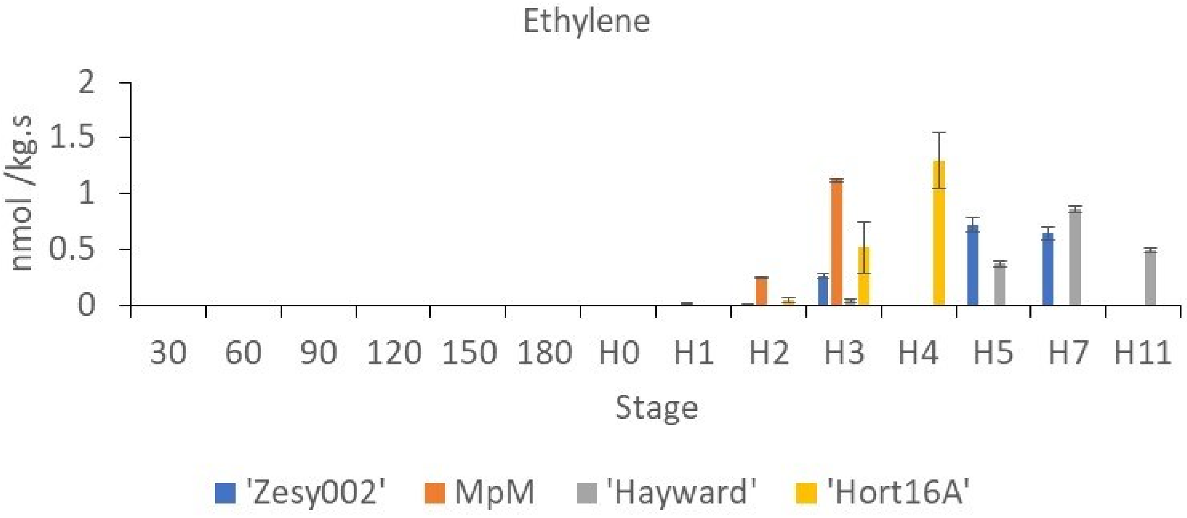
Ethylene
nmol /kg.s
2
1.5
1
0.5
0
30
60
90
120
150
180
H0
H1
H2
H3
H4
H5
H7
H11
Stage
'Zesy002'
MpM
'Hayward'
'Hort16A'
